# Probabilistic Inference of Biochemical Reactions in Microbial Communities from Metagenomic Sequences

**DOI:** 10.1371/journal.pcbi.1002981

**Published:** 2013-03-21

**Authors:** Dazhi Jiao, Yuzhen Ye, Haixu Tang

**Affiliations:** 1 School of Informatics and Computing, Indiana University, Bloomington, Indiana, United States of America; 2 Center for Genomics and Bioinformatics, Indiana University, Bloomington, Indiana, United States of America; The Centre for Research and Technology, Hellas, Greece

## Abstract

Shotgun metagenomics has been applied to the studies of the functionality of various microbial communities. As a critical analysis step in these studies, biological pathways are reconstructed based on the genes predicted from metagenomic shotgun sequences. Pathway reconstruction provides insights into the functionality of a microbial community and can be used for comparing multiple microbial communities. The utilization of pathway reconstruction, however, can be jeopardized because of imperfect functional annotation of genes, and ambiguity in the assignment of predicted enzymes to biochemical reactions (e.g., some enzymes are involved in multiple biochemical reactions). Considering that metabolic functions in a microbial community are carried out by many enzymes in a collaborative manner, we present a probabilistic sampling approach to profiling functional content in a metagenomic dataset, by sampling functions of catalytically promiscuous enzymes within the context of the entire metabolic network defined by the annotated metagenome. We test our approach on metagenomic datasets from environmental and human-associated microbial communities. The results show that our approach provides a more accurate representation of the metabolic activities encoded in a metagenome, and thus improves the comparative analysis of multiple microbial communities. In addition, our approach reports likelihood scores of putative reactions, which can be used to identify important reactions and metabolic pathways that reflect the environmental adaptation of the microbial communities. Source code for sampling metabolic networks is available online at http://omics.informatics.indiana.edu/mg/MetaNetSam/.

## Author Summary

We present a probabilistic sampling approach to profiling metabolic reactions in a microbial community from metagenomic shotgun reads, in an attempt to understand the metabolism within a microbial community and compare them across multiple communities. Different from the conventional pathway reconstruction approaches that aim at a definitive set of reactions, our method estimates how likely each annotated reaction can occur in the metabolism of the microbial community, given the shotgun sequencing data. This probabilistic measure improves our prediction of the actual metabolism in the microbial communities and can be used in the comparative functional analysis of metagenomic data.

## Introduction

Metagenomics aims to analyze the microbial communities directly extracted from their living environment, bypassing the requirements of isolating and culturing the microbes. With the recent progress of the next generation sequencing (NGS) technologies, the shotgun sequencing of a whole microbial community has become a routine exercise. As a result, the list of metagenomics studies is growing rapidly [1], [2]. This provides ample opportunities for researchers to develop new computational methods to analyze the sequences from metagenomics projects.

To understand the functional and metabolic potential of a microbial community given the sequencing data, a key analysis is to predict - from raw NGS reads or assembled contigs - protein coding genes and their functions. Functional annotations are often achieved by similarity search (using BLASTX [3], or faster tools like BLAT [4] or RAPSearch [5]) against gene families collected in the databases of biological pathways, such as Kyoto Encyclopedia of Genes and Genomes (KEGG) [6], MetaCyc [7], or SEED [8] so that biological pathways can be reconstructed from the predicted functions. Although the principle is the same, different annotation systems may use different practices: for example, the HUMAnN pipeline directly predict gene families and pathways from short sequence reads based on similarity searches [9], while MG-RAST first predicts protein coding region from short reads *de novo*, and then predicts the functions of the predicted proteins based on similarity searches [10].

Differential functions or biological pathways can be identified by comparing annotations of metagenomes, providing insights into the differences of functionality of various microbial communities [11]–[13]. For example, in recent work, the community-level metabolic networks of the microbiome were constructed from metagenomic data, and both gene-level and network-level topological differences were identified as associated with the host-based environments [14]. For quantitative analysis, the abundances of genes (often measured as the reads counts) need to be normalized according to gene lengths (more reads will be sampled from longer genes), and the quantification of pathways needs to further consider the different sizes of the pathways (*i.e.*, the number of gene families each pathway contains) and the overlaps among different pathways [15], [16].

In this paper, we present a computational method for inferring the functional activities in a metagenome on the basis of the *metabolic reactions* catalyzed by predicted genes from the dataset, instead of the genes themselves. By directly working on reactions in the context of a global network, our method is immune to the problem of pathway reconstruction caused by the overlaps between pathways - pathways are important for understanding the biological processes, however, their definition can be rather arbitrary, and the overlaps between pathways are artificially created. More importantly, our new method computes the likelihood of each reaction for all potential reactions catalyzed by predicted functions. Clearly, using all potential reactions can lead to an unfaithful estimation of the functionality of a microbial community: functional predictions are noisy and contain mistakes; on the other hand, there are genes that indeed have multiple functions [17], but not all these functions are carried out by the microbial community. Our previous approach MinPath [16], which has been incorporated in HUMAnN [9], improves pathway reconstruction for metagenomes by removing spurious pathways; MinPath, however, does not provide confidence for individual reactions inferred from metagenomic datasets.

We propose a probabilistic approach to estimate the *likelihood* of each reaction in a metagenome-scale metabolic network given predictions of enzymes. Our method computes the *marginal probability* of each reaction observed in a collection of randomly sampled subnetworks from the metagenome-scale metabolic network. In these subnetworks, for each annotated gene family, there exists at least one reaction that is carried out by the product of the gene (*i.e.* the enzyme). However, if the product of a gene is annotated to catalyze multiple reactions, some of these reactions may be excluded from the sampled subnetwork, as long as at least one of these reactions is included. We note that, according to this condition, each sampled subnetwork represents a putative reconstruction of the collective metabolic network of the metagenome, among which we assume the subnetworks containing fewer metabolites are more likely to represent the actual metabolism of the microbial community than the ones containing more metabolites. Based on this *parsimony* concept, we devised a Markov Chain Monte Carlo algorithm [18], by which we randomly sample a large set of subnetworks and estimate the likelihood of each reaction.

A microbial community adapts its collective metabolic profile to its living environment. Therefore, the similarity measure based on either protein content or metabolic activities in metagenomes can be used to cluster the metagenomes, consistent to similarity of the environments [19]. We applied our method to analyze 44 samples from several metagenomics studies: we used different measures to calculate the similarity of samples, and our results show that the distance measure based on the probability of reactions leads to the most discriminating clustering of the samples. Notably, the functional variations among metagenomes from different environmental niches cannot be fully explained by their differences in taxonomic composition, because the clustering of these metagenomes based on their metabolic taxonomic composition is not as discriminating as our method. We also show detailed comparison of the samples from two ecosystems, to demonstrate that how the probabilities of reactions can help identify important metabolic pathways that reflect the environmental adaptation of the microbial communities.

## Results

### Probabilistic Inference of Biochemical Reactions in Different Environments

From the IMG/M metagenome repository [2], we downloaded 44 metagenomic datasets, which were acquired in 10 separate metagenomics studies of different host-associated or environmental ecosystems: human and animal gut, soil, ocean, freshwater and saline lake water (Table S1). All these studies were conducted by using Illumina sequencers with massive amount of reads acquired (short reads data file size ranging from 250 MB to over 200 GB). For each sample, IMG/M provides the assembled metagenome, and the protein-coding genes are characterized with additional functional annotations, such as KEGG ortholog groups of enzymes. Based on these identified KEGG ortholog groups and the KEGG reference metabolic pathways, we constructed a metagenome-scale annotated global metabolic network for each metagenomic sample. Note that these annotated global metabolic networks contain a similar number of multi-functional enzymes (Table S1). For each metabolic network, we applied the MCMC sampling method and computed the marginal probabilities of all annotated reactions. These probabilities can be used to compare the similarity of the microbial communities in the corresponding environments.

### Clustering of Metagenomics Samples from Different Environments

We clustered the 44 samples based on different similarity measures of their enzyme contents or metabolic reactions. Five types of measures were used to estimate the distance between the metagenomics samples and compare the clustering results (for details see Methods): 1) the Bray-Curtis dissimilarity 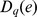 that compares the quantities of the metabolic enzymes encoded in each pair of the metagenomics samples; 2) the binary distance 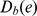 between the binary vectors representing the presence/absence of each enzyme in the metagenomes; 3) the binary distance 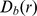 between the binary vectors representing the presence/absence of each reaction in the annotated global metabolic network based on the naive annotation of enzymes; 4) the taxonomic distance 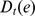 based on the phylogenetic composition of the prokaryotes involved in metabolism; and 5) the Euclidean distance 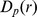 that compares the marginal probabilities of the reactions estimated by using the MCMC algorithm.

**Figure 1:**
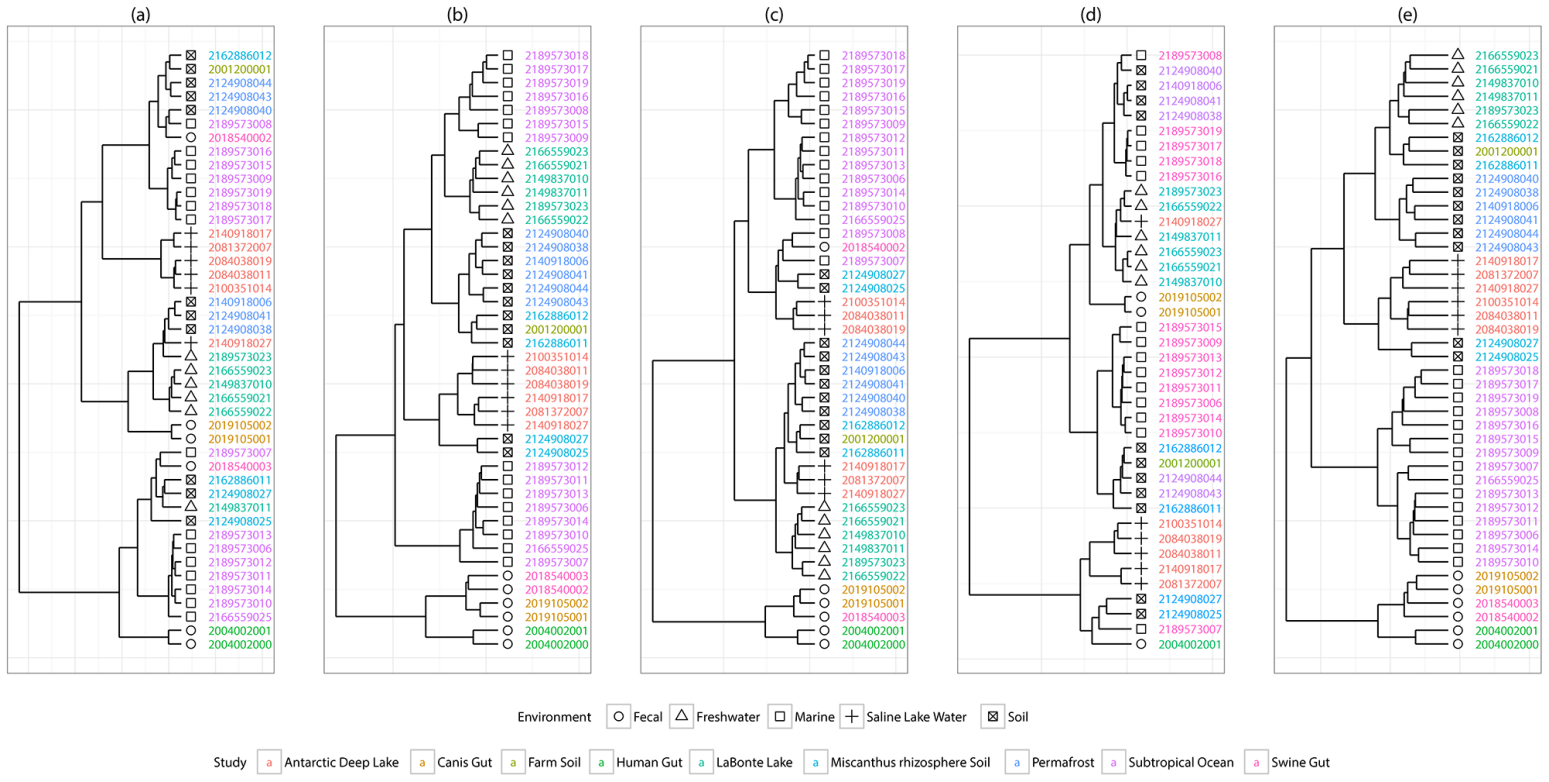
Hierarchical clustering of 44 IMG/M metagenomics samples represented in dendrograms. Five different distance measures of the metabolic patterns were used in the clustering. Sample taxon IDs are colored according to the metagenomics study. The environments of the samples are represented in shapes. (a) Clustering with distance measure 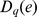 based on quantities of metabolic enzymes; (b) Clustering with distance measure 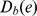 based on presence/absence of enzymes; (c) Clustering with distance measure 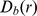 based on presence/absence of reactions; (d) Clustering with distance measure 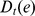 based on the taxonomic compositions; (e) Clustering with distance measure 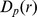 based on the likelihood of metabolic reactions. See text for details about the distance measures.

The hierarchical clustering of the metagenomics samples (for details see Method) using the five distance measures are shown in Figure 1. It is clear that the clusters created by using 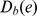 (Figure 1 (b)) and 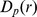 (Figure 1 (e)) are more consistent with the actual environmental similarities than the ones using the other distance measures. Between these two, the clusters generated using 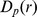 are more accurate because it can separate all metagenomic samples based on their habitats while the other method failed to. Research has shown that lake water microbial communities are highly affected by inoculation of microbes from soils [20], therefore, soil samples and lake water samples are considered to be from similar environments in previous studies [21]. This correlates well with the 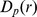-based clustering result, in which the soil samples and lake water samples are intermixed in one large cluster (Figure 1 (e)). Figure 1 (d) shows that the taxonomic composition derived from the genes involved in metabolic pathways cannot discriminate the microbial communities to their habitat groups very well, which implies that the functional similarities of the metabolisms cannot be completely attributed to the taxonomic composition of the metagenomes. The poor outcome when 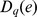 (enzyme quantities-based distance, Figure 1 (a)) is used as the distance measure indicates that prudence should be taken when using the enzyme abundances estimated in metagenome assemblies as a measure of metabolism in the microbial communities, even though it is shown to be useful in comparing relative abundance of metabolic functions in addition to binary functional reconstructions [9], [13], [14]. The poor performance of the clustering when the enzyme-coding genes are naively annotated to all the reactions (Figure 1 (c)) confirms our proposition that the naively annotated metabolic network cannot reflect the nature of the metabolic adaptation of the microbial community to its environment. The clustering results show that by computing the likelihood of the reactions occurring in the metabolism, the adaptation of the metabolism that was hidden in the global metabolic network can be revealed. This leads to a more accurate assessment of the functional similarities among the metagenomic samples.

### Comparative Functional Analysis Based on Metabolic Reactions

To investigate how the distance measure 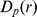 improved the clustering results compared to 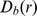, we focused our analysis on the metagenomic samples from two ecosystems: the permafrost soil samples from Alaska, and the saline lake water samples from an Antarctic deep lake. The first group of samples was collected from three different layers (two samples in each layer) in permafrost in the sediment of a creek in Alaska [22]. The second group of lake water samples was collected at six different depths ranging from 5 to 36 meters in Antarctic lakes [23]. Note that when using 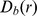 as the distance measure, 3 saline water samples were incorrectly grouped into the cluster of permafrost samples; but the distance measure 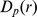 can accurately separate the samples from the two environments (Figure 1, (c) and (e)).

**Figure 2:**
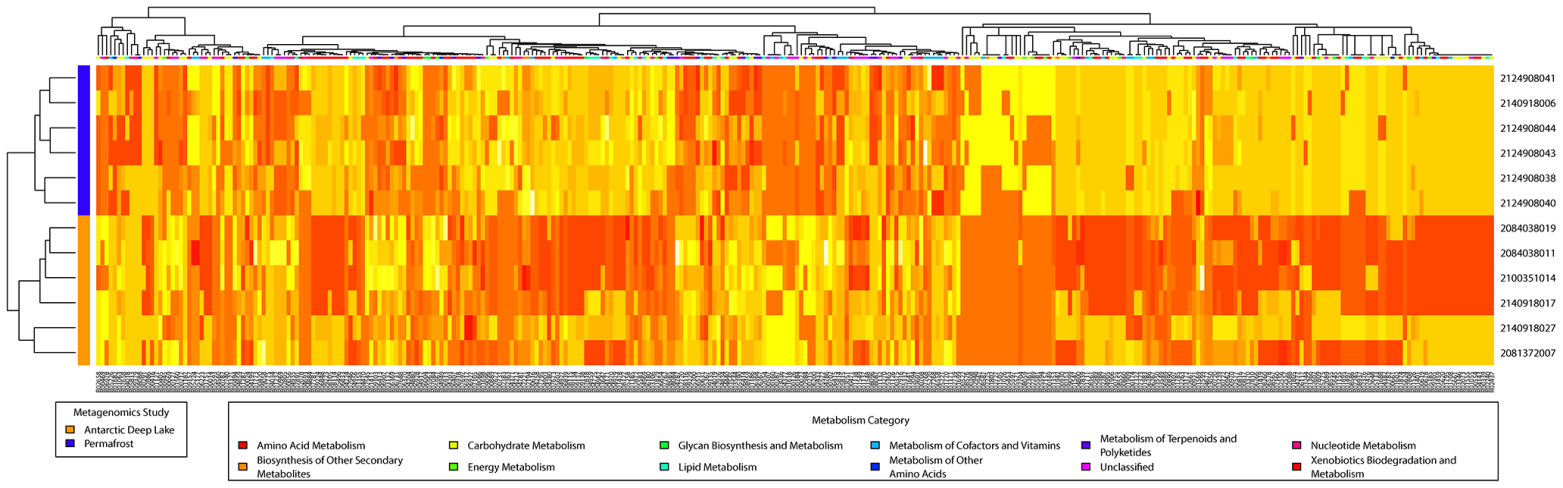
Heatmap of the reactions found to differentially occur in Antarctic deep lake and permafrost samples only by the t-test on the marginal probabilities of reactions. 338 reactions are shown in rows, and the 12 metagenomes are shown in columns. These reactions are not found to be different in the two environments if only their occurrences in the metabolic networks are compared. The two groups of metagenomics samples from two environments are separated in the clustering results. The 6 permafrost samples are grouped into 3 clusters correctly, with each cluster contains two samples from the same layer in the permafrost soil.

We used statistical tests to assess whether a metabolic reaction is differentially likely to occur in the two environments. Because the presence/absence of the reactions in the metabolic network is represented by a binary vector, we identified 110 reactions using the Fisher's exact test [24] that are statistically different (P-value
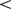
0.05) in the two groups (for details see Methods), indicating these reactions are likely to occur only in one of the two environments. We then used t-test to check if the marginal probability of each metabolic reaction is significantly different between the two groups. The t-test identified 447 reactions showing statistically different likelihoods to occur in the two environments (Table S2). The two tests agree on 109 reactions, and 338 reactions are considered to be different between these two sets of samples only by the t-test (Figure 2) on the likelihood of reactions, whereas only one reaction is detected as significantly different only by the Fisher's exact test.

**Figure 3:**
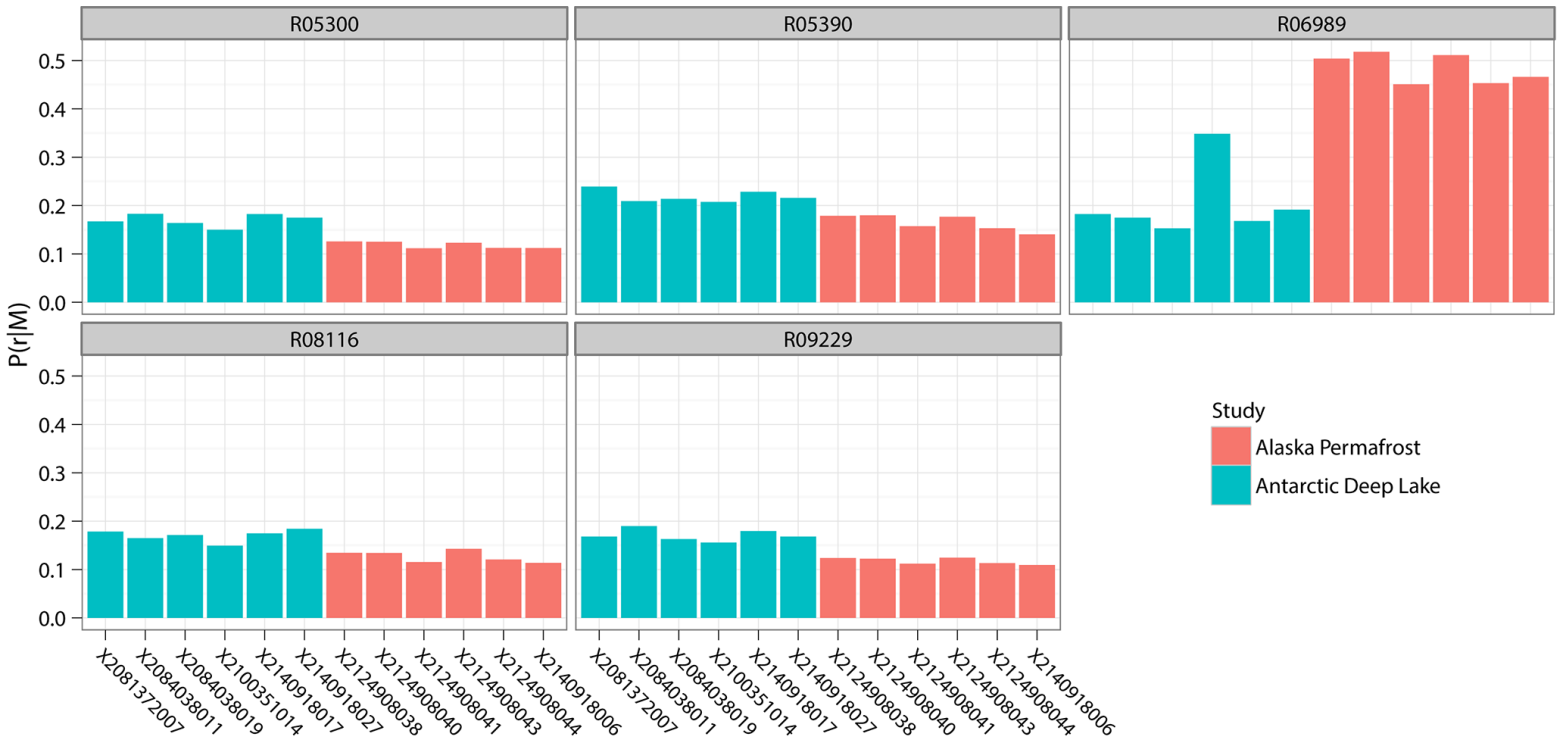
Probabilities of the 5 reactions catalyzed by muconate cycloisomerase (K01856). The difference of the probabilities of the reaction R06989 between the two groups is more significant than the other reactions.

Note that 166 of these 338 reactions are annotated to be catalyzed by one or more catalytically promiscuous enzymes in all of the metabolic networks (Table S2). In other words, there is no difference if we compare whether they exist (based on the annotation of genes) in the metabolic networks in both groups. However, the marginal probabilities of these reactions, which were assigned by the MCMC algorithm, are different among the two groups of samples, indicating these reactions show different likelihood to occur in the metabolism of the samples between the two groups. For example, one reaction in the Benzoate degradation pathway (KEGG reaction R06989) is observed in all 12 metabolic networks with different likelihoods in both environments; the reaction is on average 2.5-fold more likely to occur in the permafrost samples than in the lake water samples, if we compare their marginal probabilities (Table S2). This reaction is catalyzed by the enzyme muconate cycloisomerase (KEGG ortholog K01856), a promiscuous enzyme that also catalyzes four other reactions (Figure 3). All five reactions involve the isomerization of cis,cis-muconate and its derivatives (Figure S1). In particular, the reaction R06989, which is an important step in benzoate degradation, transforms cis,cis-muconate, which is enzymatically produced from catechol. The functions of benzoate and catechol metabolism are also found to be enriched in the permafrost microbial communities by other studies [25]. The results of the MCMC simulation show that the differences of the marginal probabilities of the other four reactions are much smaller compared to R06989. Also note that the probabilities of the five reactions are almost the same in the Antarctic lake samples, whereas, in the Alaska permafrost samples, the reaction that isomerize cis,cis-muconate (R06989) apparently has greater probabilities (Figure 3). This shows how the results of our method can be used to analyze the potential adaptation of the functions of promiscuous enzymes in different environments, which cannot be revealed when analyzing only the enzyme-encoding genes.

Another interesting observation is that the difference between the marginal probabilities of the 338 reactions can be used to correctly cluster the samples into two groups (Figure 2). In addition, if we focus on the samples extracted from Alaska permafrost, all 6 samples are correctly separated into three clusters, with each containing two samples from the same layer in the permafrost. This shows that those reactions only identified by comparing the estimated marginal probabilities contain the critical information in the metabolic adaptations of these samples to their environments.

**Figure 4:**
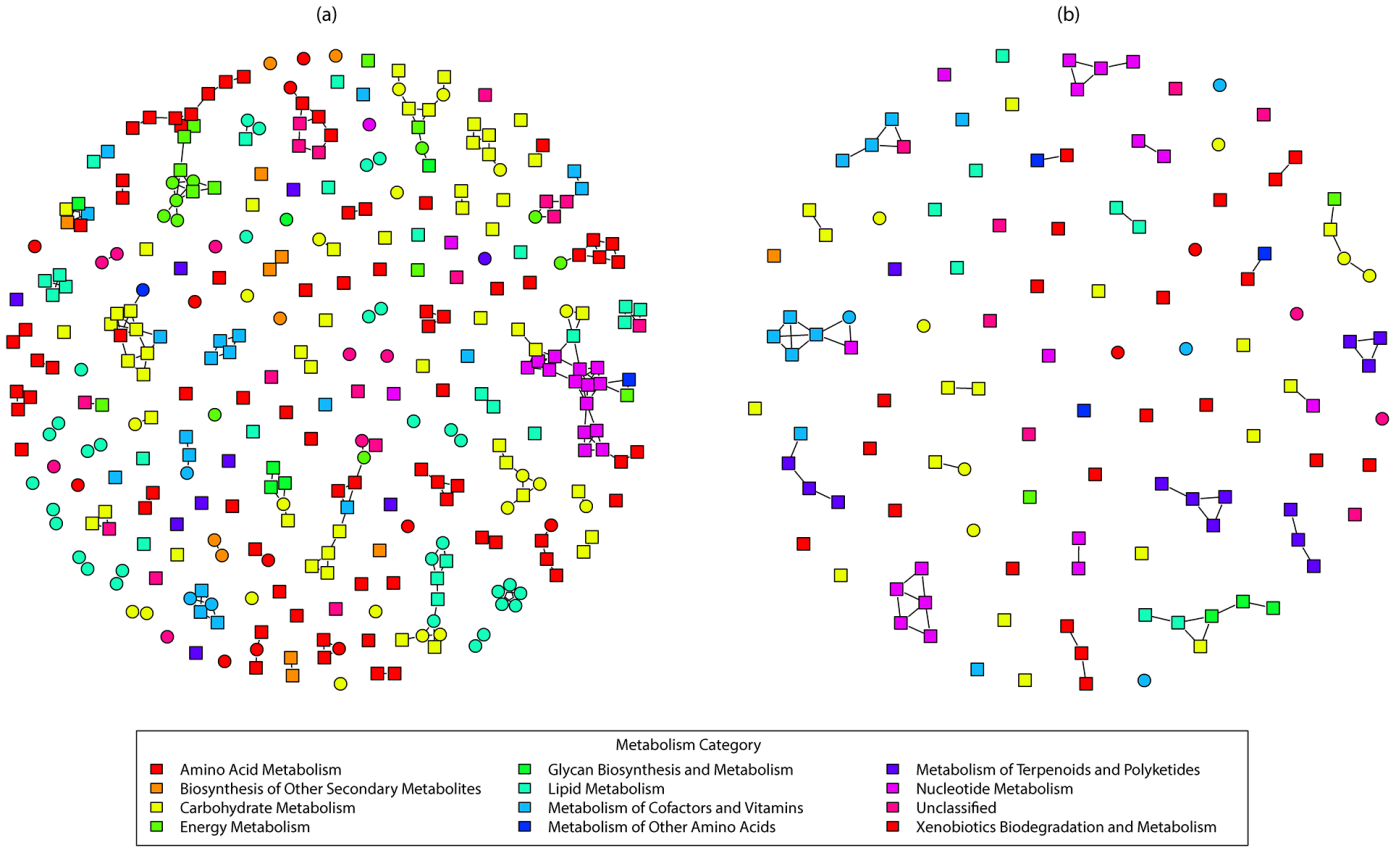
Network of reactions that are different in the two environments. Each vertex represents a reaction. An edge is connected between two vertices if the two reactions share one or more metabolites. Square shaped vertices represent the reactions discovered to be different by using t-test on marginal probabilities, but not different when using the Fisher's test on the enzyme occurrences; Circle shaped vertices represent the reactions considered to be different in both statistical tests. (a) 327 reactions with higher marginal probabilities in Alaska permafrost samples; (b) 120 reactions with lower marginal probabilities in Alaska permafrost samples.

Among those 447 reactions that are identified to be different by the t-test on the marginal probability, 327 reactions show higher marginal probabilities to appear in the Alaska permafrost samples than in Antarctic deep lake samples. The remaining 120 reactions show lower probabilities in the Alaska permafrost samples than the Antarctic deep lake samples (Table S2). We built two networks using these two sets of reactions. In these networks, vertices represent the reactions, and a pair of reactions is connected by an edge if there are one or more common metabolites in the two reactions(Figure 4). The connected components in these networks represent *chains* of metabolic reactions that can be considered to have significant higher probabilities to occur in the environment of one group compared to the other.

**Figure 5:**
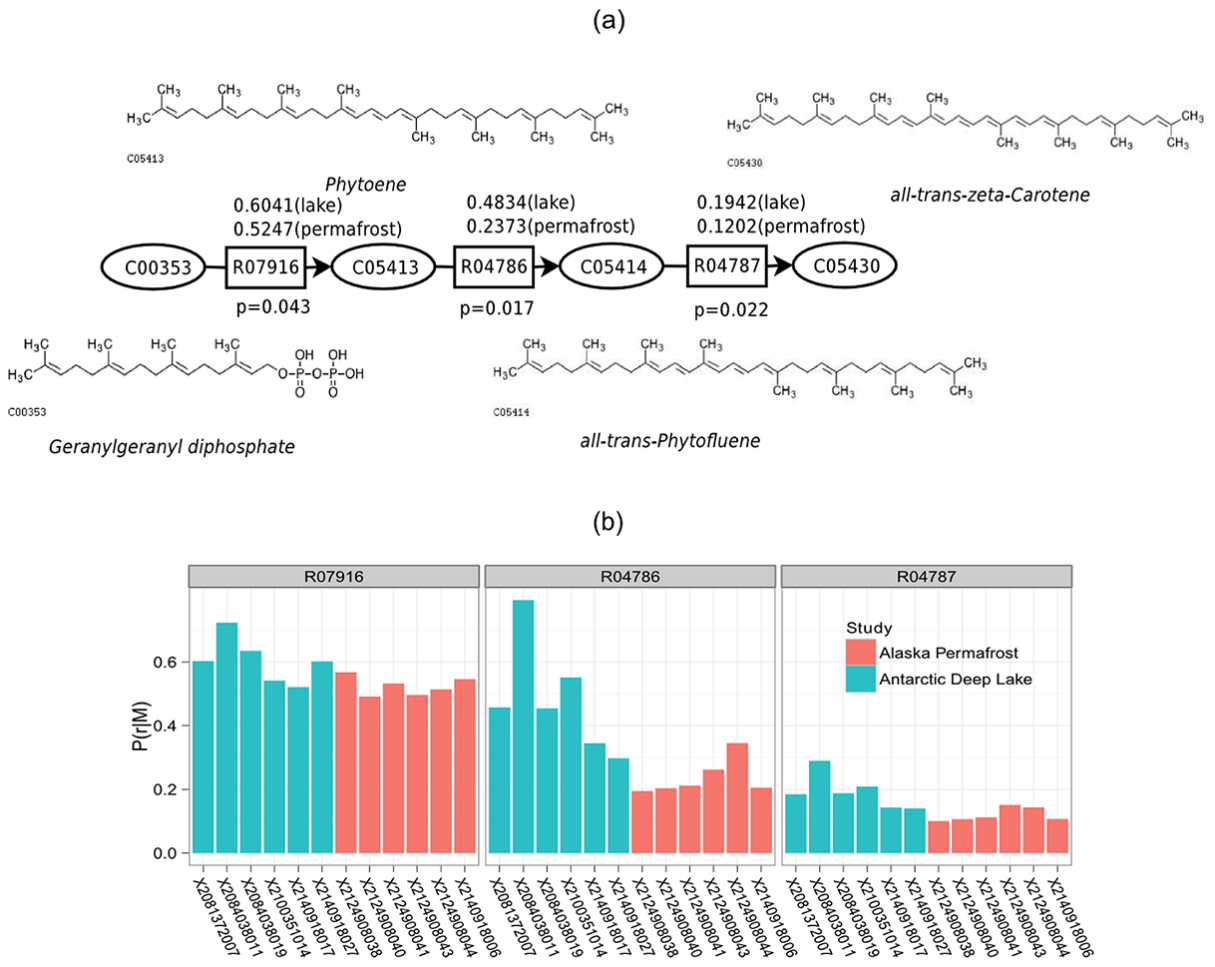
R07916-R04786-R04787 reaction chain. It is part of the beta-carotene biosynthesis module, and is observed to be different in the metagenomic samples from the two environments, Antarctic lakes and Alaska permafrost. (a) Reactions and metabolites. Values above the reaction boxes are the average marginal probabilities of the reactions in the two groups. Values under the reaction boxes are the p-values from the t-test. (b) The marginal probabilities of the reactions in different samples.

Several interesting chains of reactions were revealed in both networks. For example, the chain R07916-R04786-R04787 (R07916, R04786, R04787 are KEGG reaction IDs) has a higher probability to occur in the metabolism of microbial communities from Antarctic deep lakes (Figure 5), which is a part of the beta-carotene biosynthesis module belonging to the carotenoid biosynthesis pathway. Carotenoids are essential metabolites for photosynthetic bacterial because they provide photo-protection and accessory light harvesting [26]. The bacterial community in fresh water is known to carry out photosynthetic activities even in deep water. The Antarctic deep lake metagenomics study also revealed trace of photosynthetic microorganisms in their samples [23]. Therefore, it is not surprising to observe that photosynthesis related pathway modules have a higher likelihood to occur in the deep lake microbial communities than in the permafrost soil samples, which exist in an environment deprived of light. Several chains of reactions were found to have higher probabilities to occur in permafrost samples, among which were chains in methanogenesis, and keratan sulfate degradation. Slow rates of methanogenesis by cold-adapted methanogens occur in permafrost and active layer soils [22], and keratan is regarded as a carbon source for certain bacteria isolated from soil [27]. Note that these chains all contain reactions that are identified only by comparing the reaction marginal probabilities but not the enzymes or the existence of reactions. Some chains even contain only reactions that are identified by comparing the reaction marginal probabilities (Figure 4). Therefore, our method successfully expands the horizon of discovering important pathways that contain critical information of the adaptive metabolism of microbes.

## Discussion

In this paper, we focus on the analysis of metagenomics samples based on the metabolic reactions annotated to be catalyzed by the predicted genes in the metagenomes. We proposed a method that assigns marginal probabilities to reactions to estimate the likelihood of the reactions to occur in the metabolism of a microbial community. The Markov Chain Monte Carlo (MCMC) sampling algorithm establishes a framework that can be used to study the different aspects of metabolic networks. The subnetwork universe can be sampled by the MCMC algorithm with different constraints based on various assumptions. For example, in recent work, flux balance analysis (FBA) is used to constrain the viability of the sampled genome-scale metabolic networks in a MCMC based method [28].

The marginal probabilities are assigned by our method to reactions that are catalyzed by catalytically promiscuous enzymes. As shown in the results, the reactions occurring in a microbial community are a better representation of the metabolism in the community, because one reaction may be catalyzed by different enzymes encoded by different microbial organisms in the community. An extension of this method is to compare the likelihood of the reactions to be catalyzed by the same enzyme and allow us to investigate how promiscuous enzymes function in different environments.

By applying the parsimony assumption, our method successfully takes several intrinsic properties of the metabolic network into consideration. It should be noted that this method indirectly favors highly connected metabolic networks, where the number of non-terminal metabolites that can be produced and consumed by the microbial community is maximized. Similar assumptions have been used in other studies. For example, in the metabolic network reconstruction, gaps in metabolic paths are usually filled to decrease the number of isolated reactions or metabolites [29]. Notably, this assumption is particularly practical for the study of the metabolism in a microbial community rather than individual microbial organisms, because the microbes living in the same environment likely co-evolved into a condition under which microbes can make use of the metabolites from other microbes and only a small number of metabolites are required from the external environment by the whole community.

We note that our method lacks the compartmentalization of the biochemical reactions and the resolution of individual species. In previous studies, multi-species models have been applied to investigate the interactions within the community or between host and microbiomes [30]. In comparison, when studying the system-level behavior of the whole microbial communities, researchers often treat the microbial communities as individual adaptive organisms (also referred as *supra-organism*), ignoring the boundaries between species altogether [14], [31], [32]. In this study, we take a similar approach, which allows us to investigate the collective metabolic behavior of the microbial communities. This approach is also a necessity because genomic information is not available for all the species in the community and methods for decomposing complex metagenomic samples into compartmentalized organelles/prokaryotic cells are yet to be developed.

There are, however, several limitations of the method that are worth noting. In our metabolic network definition, the reactions are considered to be indirect, which indicate that all reactions are reversible. However, conditions in the cell are often such that it is thermodynamically infeasible for flux of reactions to flow in certain direction so the reaction becomes irreversible. Therefore, there might be *dead-end metabolites* in the network, the metabolites that are not the product of the other reactions or are not used by other reactions as substrates. In our model, they are misinterpreted as the metabolites that connect two reactions, which could decrease the accuracy of the reaction and pathway annotated by our method. Another issue is that this method does not consider the abundance of enzymes predicted from the metagenomic sequences. We observed large variance in the abundance of the same enzyme among samples as well as pairs of enzymes that share same metabolites as substrates or products. We are working on a revision of our current method to take the abundance of enzymes into account.

## Methods

### Problem Formulation

We start the problem formulation with a formal definition of a metabolic network.

#### Definition 1

*A reference metabolic network is a labeled undirected graph* 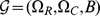 *where* 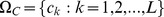 *represents a set of* 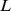 *vertices, each labeled by a metabolite (compound), and* 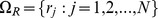 *represents a set of* 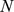 *edges, each labeled by a biochemical reaction. The graph topology is represented in a matrix form* 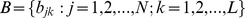,
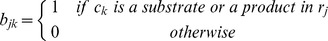



We then annotate the enzymes corresponding to the predicted genes in a metagenomic dataset on the reference metabolic network (in the database such as the KEGG [6]), and define the *annotated global metabolic network* as follows.

#### Definition 2

*Considering the set of* 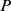 *metabolic enzymes annotated in a metagenome as* 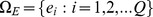, *the reactions in the reference network that are annotated by enzymes are represented as a matrix* 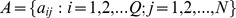


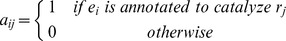



*We call* 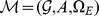 an annotated global metabolic network *of the metagenome*.

In the annotated metabolic network, each reaction is annotated to be catalyzed by one or more enzyme, whereas each enzyme is *naively* annotated with all of its putative catalytic functions. The annotated metabolic network provides a global view of the metabolic activities encoded in a metagenome. Note that in an annotated network, there are usually a substantial number of catalytically promiscuous enzymes that are annotated to catalyze more than one reaction. The promiscuity of the enzymes is due to the factors such as the environmental conditions such as pH and temperature, and under certain circumstances, a promiscuous enzyme more likely catalyzes one of its annotated reaction than the others [17]. Altogether, the annotated global metabolic network represents the universe of all metabolic profiles; but it does not reflect the adapted metabolism to certain environmental conditions because all functions of the promiscuous enzymes are considered to occur equally likely.

Below we introduce the concept of a subnetwork of the annotated global metabolic network as a representation of one putative metabolic profile of the metagenome. A subnetwork consists of a subset of reactions, metabolites and enzymes from the global network; a subnetwork is defined as *valid* if each predicted enzyme is annotated to catalyze at least one reaction in the subnetwork. Their formal definitions are given as follows:

#### Definition 3

*Given the annotation* 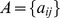 *on a gene set* 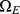 *in an annotated metabolic network* 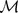, *we define an annotated metabolic subnetwork* 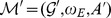, *where* 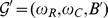 *is a subgraph of the metabolic network* 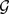 *(i.e.,* 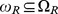, 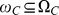, *and* 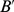 *is a submatrix of* 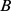 *),* 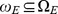, *and* 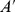 *is a submatrix of* 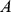.

#### Definition 4

*An annotated metabolic subnetwork* 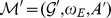 *of a annotated global metabolic network* 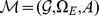 *is called valid, if* 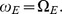


The set of valid subnetworks from an annotated global metabolic network constitutes the universe of *valid subnetworks* 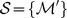. Within the scope of our study, the probability of a subnetwork in the universe 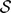 is the likelihood of the subnetwork to represent the real metabolism of the metagenome, denoted as 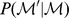. Assuming the probability distribution of 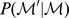 is known, we can calculate the marginal probability of each reaction given the annotated global metabolic network 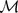 based on the Bayes' theorem (equation (1)):
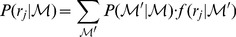
(1)where 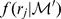 is the probability of a reaction 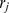 to occur in the subnetwork. Because the configuration of the subnetwork is already known, it can be represented as an indicator function,
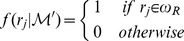



Due to the complexity of metabolic networks, it is intractable to compute 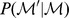, because the subnetwork universe is huge – the total number of potential subnetworks is 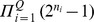, where 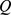 is the number of enzymes in the annotated global metabolic network, and 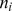 is the total number of reactions that enzyme 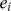 catalyze. Given the number of multi-functional enzymes that an annotated global metabolic network contains, it's not tractable to enumerate all potential subnetworks to compute 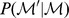. Here, we resort to a Markov Chain Monte Carlo (MCMC) algorithm to sample subnetworks from the subnetwork universe 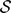, and compute the marginal probabilities based on the subnetwork samples.

### The Parsimony Assumption and Inequality of the Likelihood of Metabolic Subnetworks

Before we discuss the details of the MCMC algorithm, we introduce an inequality of likelihood of the metabolic subnetworks based on a parsimony assumption. We observe that within microbial communities, metabolic enzymes work collectively to catalyze a series of reactions to transform some compounds that are available in their living environment into other compounds that can be utilized to maintain their cellular functions. Although the enzymes act on different substrates and products, the products of some reactions are usually used as substrates in the subsequent reactions, resulting in a sequence of reactions devising the complex but efficient metabolic network. The adaption of a living microbial organism to its living environment often leads to a unique and nearly optimal metabolic network, in which only a small number of *necessary* compounds need to be taken from its environment, while the other compounds can be synthesized through metabolic reactions inside the microbial community. Based on these observations, we adopt a parsimony assumption: the valid subnetworks involving fewer compounds are more likely to represent the metabolism of the microbial community.

Equivalently, given two valid subnetworks 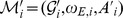 and 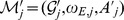 of the same annotated global network 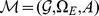, where 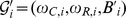 and 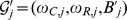, if
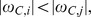
then
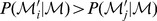
(2)


### Markov Chain Monte Carlo (MCMC) Sampling Based on the Parsimony Assumption

We construct a Markov Chain (MC) of annotated subnetworks to sample the valid annotated metabolic subnetwork in the universe 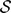 and estimate the marginal probabilities of reactions. At each step of the random MC walk, a new metabolic subnetwork is generated by inserting a new reaction to the current subnetwork, or by deleting an existing reaction from the current subnetwork. We repeat the insertion/deletion until a valid new subnetwork is generated. The transition from the current subnetwork to a new valid subnetwork is accepted or rejected based on the parsimony inequality in equation (2). If the number of metabolites found in the new subnetwork is smaller than the number of metabolites in the current subnetwork, we accept the transition to the new subnetwork; otherwise, we accept the transition with a probability 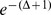 (called the *candidate probability*), where 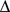 is the difference between the number of metabolites in the new and the current subnetwork.

It is straightforward to show that this type of transition is *ergodic*, *i.e.*, any pair of subnetworks can be connected by a finite series of such transitions. The candidate probability ensures that the random walk samples the subnetworks based on the parsimony assumption, as defined in equation (2). According to the Metropolis-Hastings rule [33], the candidate probability also ensures the subnetwork samples are drawn from a probability distribution that is proportional to the likelihood of the subnetwork in the subnetwork universe 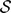.

**Figure 6:**
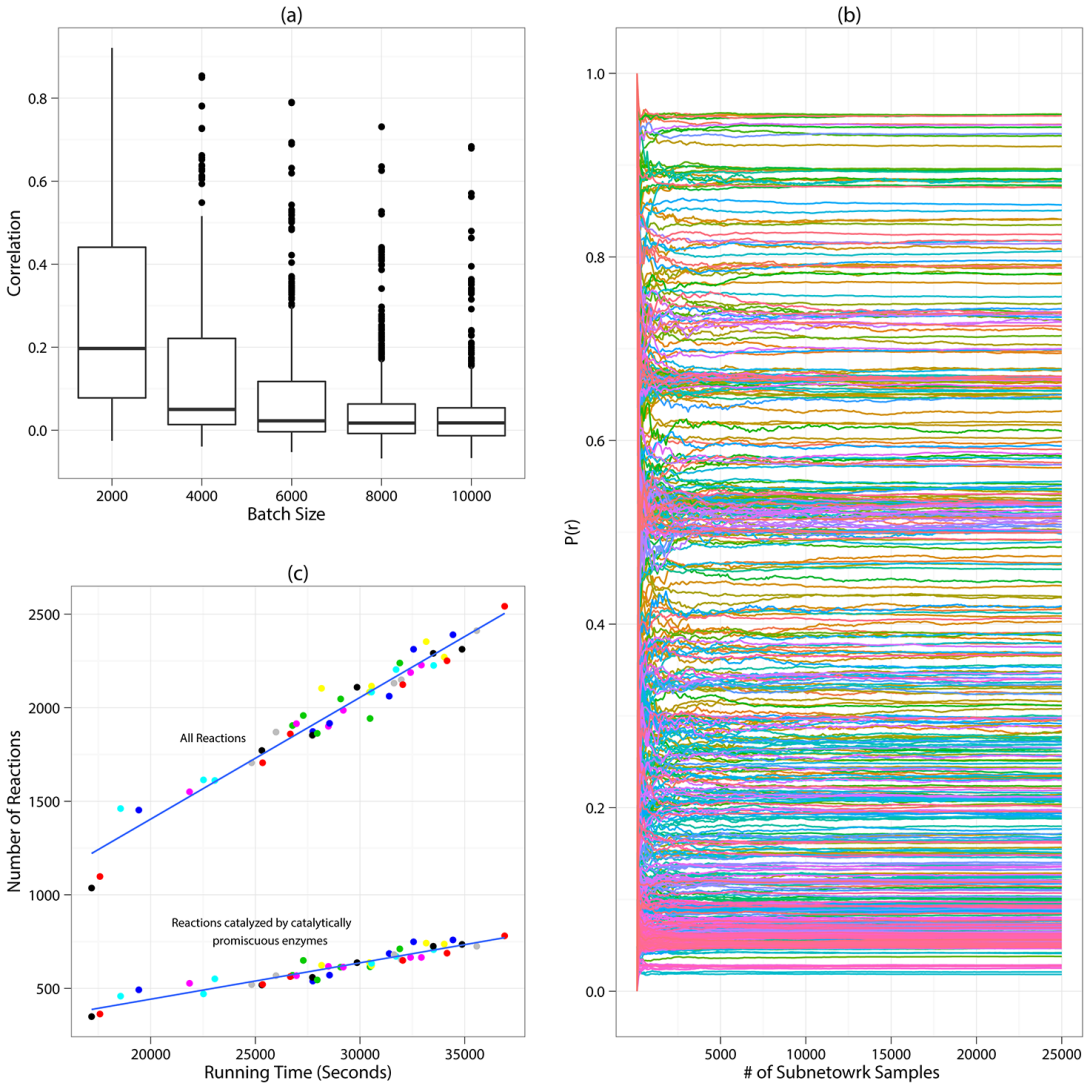
Properties of the Markov chain. (a) Correlations of the probability of reaction in consecutive subnetworks sampled from the Markov chain. As the batch size in subsampling increases, the correlation decreases and become insignificant (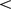
0.1) for most reactions when batch size is set to 10,000. (b) Ergodic averages of the marginal probability 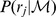 for all reactions catalyzed by promiscuous enzymes in a metagenome. (subsampling with batch size = 10,000) (c) Running time of the Markov chain of global metabolic networks of various sizes for 250 million iterations. Top are the total numbers of reactions in each sample. Bottom are the numbers of reactions that are catalyzed by catalytically promiscuous enzymes.

**Figure 7:**
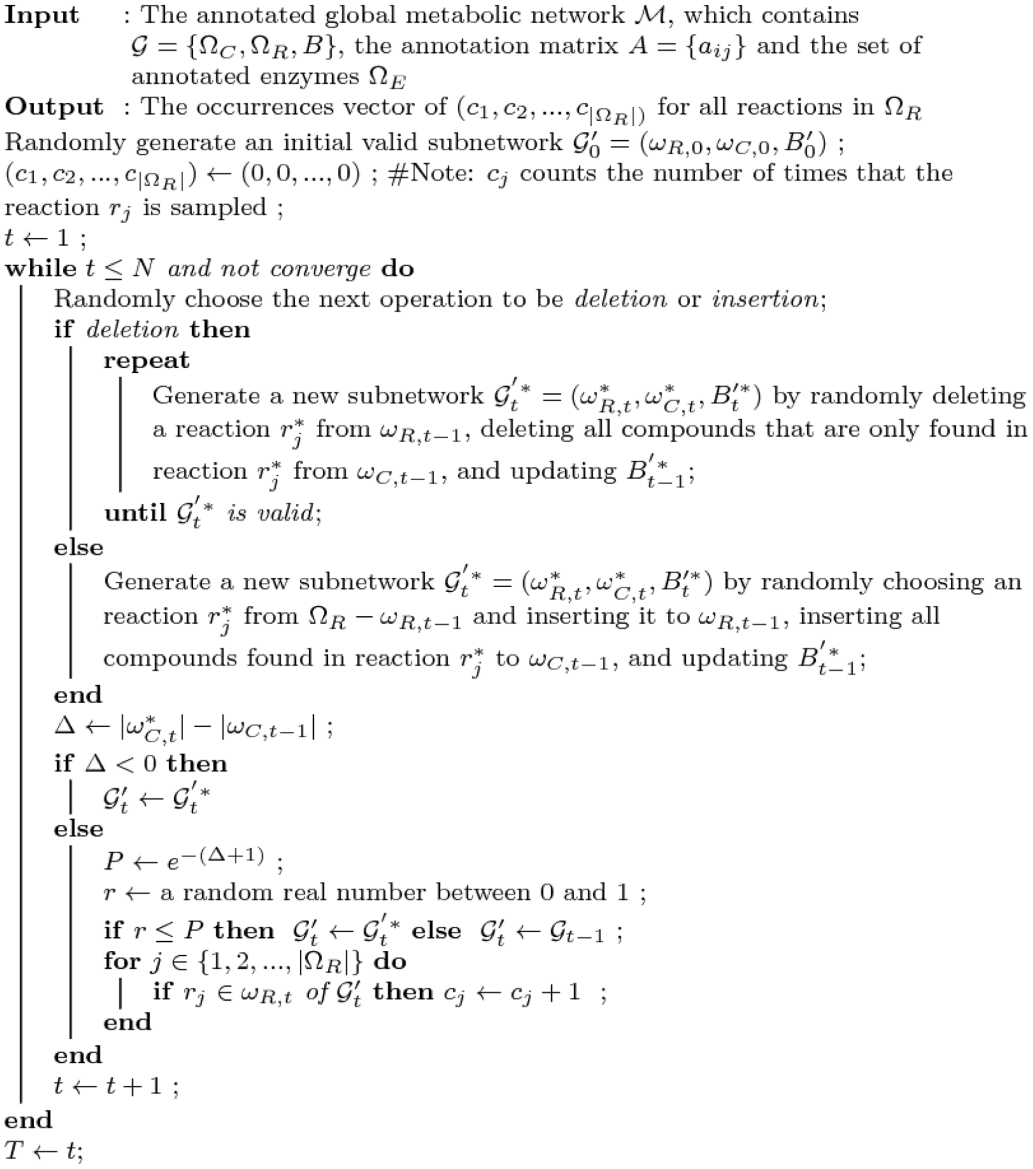
Metropolis-Hastings Algorithm.

The number of variables we are trying to estimate equals the number of reactions that are catalyzed by promiscuous enzymes. Metabolic networks constructed from metagenomic data normally contain hundreds of such reactions. Here we discuss several methodological issues of the Markov chain caused by the large number of variables in the sampling universe. First, every step in the random walk only changes the state of at most one reaction, therefore, the correlation between the two consecutive sampled subnetworks is obviously large. We use the *subsampling* (also called *batch sampling*) technique to reduce the correlation and to approximate independence between the successive samples of the Markov chain [18]. We subsample from the Markov chain with a deterministic batch size 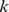, meaning that we consider only one subnetwork from every 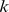 sampled subnetworks. As shown in Figure 6 (a), when 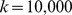 for a network containing 362 promiscuous enzymes (1183 enzymes in total), subsampling almost completely eliminates the correlation of successive sampled subnetworks. Another issue is the acceptance rate of the transitions in the Markov chain, which is the ratio of the number of accepted transitions from the current subnetwork to the new subnetwork, over the number of proposed transitions. This ratio was shown to affect the convergence pattern of the Markov chain [18], and it's heuristically recommended to be controlled to close to 1/4 for models with high dimensions [34]. The acceptance rate is affected by the candidate distribution, which in our model has the exponential distribution form 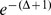, where 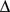 is the difference between the number of metabolites in the new subnetwork and the current subnetwork. We choose the candidate distribution in this form because it can restrict the acceptance rate of our random walk approximately within the range of 0.20 to 0.24. Note that because we only sample the valid subnetworks, the rejected invalid subnetworks in our algorithm (Figure 7) are not counted when calculating the acceptance rate. Furthermore, it requires a large number of samples from the subnetwork universe 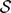 for an accurate estimation of the marginal probability. This, in addition to the requirement of subsampling to avoid high correlation between the samples, requires a careful monitoring of the convergence of the Markov chain. We examine the ergodic average of the estimated marginal probability 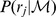 for all reaction 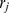 in a metabolic network of 609 reactions that are catalyzed by multi-functional enzymes, and heuristically determine that with subsampling of batch size 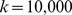, the Markov chain converges after 10,000 subnetworks are sampled (Figure 6 (b)). To improve the convergence of our Markov chain, in practice, we choose to discard samples from the first 10 million steps of the Markov chain, *i.e.*, the *burn-in period*, to make the random walk to start from a better point in the subnetwork probability space 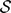. Last but not least, because of the requirements of large batch size in subsampling, low acceptance rate, and large amount of samples, the running time of the Markov chain requires special examination. We find that when we control all these factors, the running time is linearly correlated to the number of reactions in the subnetwork, or in some sense the size of the subnetwork (Figure 6 (c)). For a large network with over 1,200 reactions, of which more than 600 are catalyzed by multi-functional enzymes, the algorithm can finish 250 million iterations in approximately 10 hours.

The formal Metropolis-Hastings algorithm is shown in Figure 7.

After sampling, we can compute the estimated marginal probability 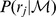 by
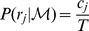
(3)for 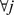 such that 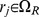, where 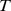 is the total number of sampled valid subnetworks.

In addition to the marginal probability 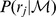, we can also extract a subnetwork sample with the maximum likelihood in the samples, which is the subnetwork with the smallest number of compounds.

### Distance Measures, Clustering Algorithm and Statistical Tests

For any pair of metagenomes 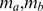, the Bray-Curtis dissimilarity is 
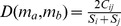
, where 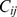 is the sum of the lesser value for only those enzymes in common in both samples. 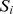 and 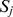 are the total number of enzymes counted in both samples [35]. In this paper, this distance is denoted as 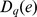. The quantities of enzymes in each metagenomic dataset were obtained from IMG/M, computed based on the number of assembled contigs aligned to each family of enzymes. The Jaccard distance is used to estimate the binary distance of the samples, based on enzymes (denoted as 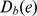) and reactions (denoted as 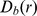). We also computed the Euclidean distance of reactions 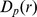, which is based on the marginal probabilities of the reactions.

The taxonomic distances, denoted as 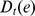 were calculated in several steps: for each metagenome downloaded from IMG/M, to ensure the comparison is based on the organisms involved in the collective metabolic processes, we removed the genes that are not annotated with a metabolic function in the IMG/M KEGG ortholog group annotations. Then we used BLAST (version 2.1.18) to search the genes against the KEGG genes database (E-value 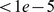). From the BLAST results, we built a phylogenetic tree by gathering the genome of the top hits and mapping them to the Greengenes core set [36]. Using the phylogenetic trees, we calculated the pairwise taxonomic distances between samples with unweighted Fast UniFrac method [37], [38] (PyCogent [39] version 1.5.3), a metric that measures the phylogenetic relatedness of whole communities and has been widely applied in studies to compare taxonomic differences between complex microbial communities [40].

We used the Ward's minimum variance method as the linkage criteria in our hierarchical clustering, which tries to minimize the total within-cluster variance. Note that when applying other common linkage criteria in the hierarchical clustering, even though the performance varies, the order of performances using the four distance measure was still observed.

Fisher's exact test [24] is used to determine if there are nonrandom associations between two binary variables. For each reaction, we used Fisher's test to compare its presence/absence in the two groups of samples from the two environments. The p-value gives the exact probability of observing the particular ratio of the presence/absence of the reaction in the samples from the two environments, on the null hypothesis that the chances of the reaction to exist in both environments are the same. We consider reactions with p-value
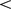
0.05 as ones that have significant different probability to be found in the two environments. We also used Fisher's test in comparing the binary representation of each enzyme in the two environments.

We used the independent two-sample t-test to determine whether the quantities of enzymes are statistically different in two environments under the assumptions that these quantities are independent and normally distributed, and the distributions of the quantities in the two groups of samples have the same variance. So for each enzyme, we consider its quantities in samples in the two environments as two groups of values, and the t-statistic determines whether the means of the two groups of values are different. Similarly, we also used t-test for analyzing whether each reaction has a different probability in the two environments.

### Implementation of MCMC Sampling

The MCMC sampler of metabolic networks is implemented in Java, and based on the HYDRA MCMC library [41]. Running time of the program is dependent on the size of the network and the configuration of the MCMC sampler, including the subsampling size and burn-in period (Figure 6). For a large network with about 2,500 reactions, the sampling takes approximately 10 hours on a dual core Dell Latitude laptop, and requires a small amount of memory (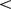
50 MB). Source codes can be downloaded at http://omics.informatics.indiana.edu/mg/MetaNetSam/.

## Supporting Information

Figure S1 **Reactions catalyzed by muconate cycloisomerase (KEGG Orothlog K01856).** All five reactions are found in all samples in the metagenomics samples from the Antarctic deep lake and Alaska permafrost studies. KEGG reactions: (a) R05300; (b) R05390; (c) R06989; (d) R08116; (e) R09229.
(PDF)

Table S1 **IMG/M metagenomics samples used in our study.** Data were downloaded on 4/10/2012.
(PDF)

Table S2 **Reactions in Antarctic deep lake and Alaska permafrost samples.** The first p-value is from the Fisher's test that checks if the occurrences of the reactions in the two groups of samples are different. This p-value equaling 1 means the reaction occurs in all the samples in the two groups. The second p-value is the t-test based on the marginal probability of the reactions. The last two columns are the average marginal probabilities of the reactions in the two groups of samples.
(PDF)
