# Supplementary material for: Probabilistic Inference of Biochemical Reactions in Microbial Communities from Metagenomic Sequences
Source: PLoS Comput Biol. 2013 Mar 21;9(3):e1002981. doi: 10.1371/journal.pcbi.1002981 (PMC3605055; doi:10.1371/journal.pcbi.1002981)
Supplement: Figure S1 — Reactions catalyzed by muconate cycloisomerase (KEGG Orothlog K01856). All five reactions are found in all samples in the metagenomics samples from the Antarctic deep lake and Alaska permafrost studies. KEGG reactions: (a) R05300; (b) R05390; (c) R06989; (d) R08116; (e) R09229. (PDF) [file pcbi.1002981.s001.pdf]

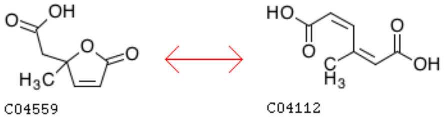

(a)

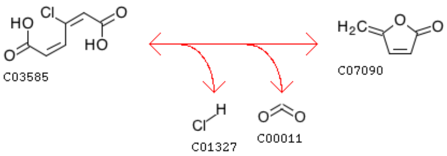

(b)

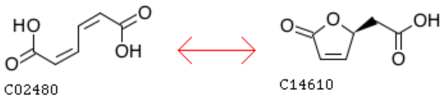

(c)

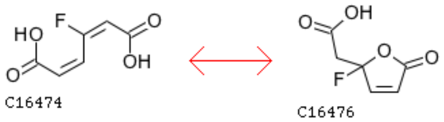

(d)

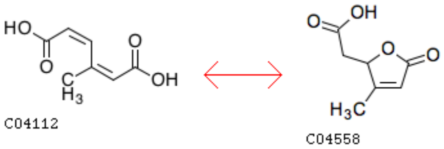

(e)

Figure S1. Reactions catalyzed by muconate cycloisomerase (KEGG Orothlog K01856). All five reactions are found in all samples in the metagenomics samples from the Antarctic deep lake and Alaska permafrost studies. KEGG reactions: (a) R05300; (b) R05390; (c) R06989; (d) R08116; (e) R09229.
