# Supplementary material for: Probabilistic Inference of Biochemical Reactions in Microbial Communities from Metagenomic Sequences
Source: PLoS Comput Biol. 2013 Mar 21;9(3):e1002981. doi: 10.1371/journal.pcbi.1002981 (PMC3605055; doi:10.1371/journal.pcbi.1002981)
Supplement: Table S1 — IMG/M metagenomics samples used in our study. Data were downloaded on 4/10/2012. (PDF) [file pcbi.1002981.s002.pdf]

**Table S1. IMG/M metagenomics samples used in our study.** Data were downloaded on 4/10/2012

| Taxon ID | Sample Name | Metagenomics Dataset | # of Enz | # of Cpd | # of Rxn | # of Rxn by CPE[1] | Running Time(ms) | Acceptance Rate |
|---|---|---|---|---|---|---|---|---|
| 2004002000 | Human Gut Community Subject 7 | Fecal microbiome of Human from distal gut of healthy adults | 703 | 1110 | 1036 | 349 | 17178349 | 0.236 |
| 2004002001 | Human Gut Community Subject 8 | Fecal microbiome of Human from distal gut of healthy adults | 733 | 1152 | 1098 | 363 | 17573011 | 0.238 |
| 2018540002 | Amitermes wheeleri | Fecal microbiome of Swine from Yorkshire Ohio | 1276 | 1931 | 1942 | 616 | 30484629 | 0.226 |
| 2018540003 | Sample 266 | Fecal microbiome of Swine from Yorkshire Ohio | 950 | 1517 | 1453 | 492 | 19433642 | 0.230 |
| 2019105001 | sample 1 | Fecal microbiome of Canis familiaris | 1122 | 1621 | 1611 | 551 | 23064458 | 0.233 |
| 2019105002 | sample 2 | Fecal microbiome of Canis familiaris | 1064 | 1584 | 1551 | 527 | 21855180 | 0.234 |
| 2081372007 | Freshwater microbial communities from Antarctic Deep Lake, sample 24m 3.0um (24 m 3.0 um Sept 2010 combined) | Freshwater microbial communities from Antarctic Deep Lake | 1298 | 2138 | 2104 | 624 | 28159486 | 0.218 |
| 2084038011 | Freshwater microbial communities from Antarctic Deep Lake, sample 24m 0.1um (24 m 0.1 um 454 only) | Freshwater microbial communities from Antarctic Deep Lake | 1046 | 1815 | 1705 | 520 | 24823466 | 0.216 |
| 2084038019 | Freshwater microbial communities from Antarctic Deep Lake, sample 5mRS 0.1um (5 mRS 0.1um 454 only) | Freshwater microbial communities from Antarctic Deep Lake | 1057 | 1851 | 1771 | 518 | 25316272 | 0.215 |
| 2100351014 | Freshwater microbial communities from Antarctic Deep Lake, sample 13m 0.1um (13m 0.1um 454 only) | Freshwater microbial communities from Antarctic Deep Lake | 1051 | 1796 | 1705 | 521 | 25349431 | 0.218 |
| 2140918017 | Freshwater microbial communities from Antarctic Deep Lake, sample 24m 0.8um (24 m 0.8 um 454/Illumina combined Jan 2011) | Freshwater microbial communities from Antarctic Deep Lake | 1255 | 2068 | 2047 | 614 | 29074012 | 0.220 |
| 2140918027 | Freshwater microbial communities from Antarctic Deep Lake, sample 36m 3.0um, 0.8um, 0.1um pool (HWGG+HTSY Jan 2011) | Freshwater microbial communities from Antarctic Deep Lake | 1547 | 2327 | 2390 | 759 | 34436517 | 0.225 |
| 2149837010 | Fresh water microbial communities from LaBonte Lake, Laramie, Wyoming, sample from pre-bloom (pre-bloom) | Fresh water microbial communities from LaBonte Lake | 1424 | 2197 | 2226 | 707 | 33518361 | 0.223 |
| 2149837011 | Fresh water microbial communities from LaBonte Lake, Laramie, Wyoming, sample from post-bloom (post-bloom) | Fresh water microbial communities from LaBonte Lake | 1251 | 2006 | 1986 | 613 | 29202833 | 0.221 |
| 2166559021 | Fresh water microbial communities from LaBonte Lake, Laramie, Wyoming, sample from peak-bloom 2 (Peak bloom metagenome 2) | Fresh water microbial communities from LaBonte Lake | 1522 | 2294 | 2353 | 742 | 33164141 | 0.225 |
| 2166559022 | Fresh water microbial communities from LaBonte Lake, Laramie, Wyoming, sample from algal/cyanobacterial bloom material peak-bloom 1 (algal/cyano bloom peak-bloom 1) | Fresh water microbial communities from LaBonte Lake | 1488 | 2366 | 2412 | 725 | 35579822 | 0.220 |

[1] Catalytically Promiscuous Enzyme

**Table S1. IMG/M metagenomics samples used in our study. (continued)**

| Taxon ID | Sample Name | Metagenomics Dataset | # of Enz | # of Cpd | # of Rxn | # of Rxn by CPE | Running Time(ms) | Acceptance Rate |
|---|---|---|---|---|---|---|---|---|
| 2166559023 | Fresh water microbial communities from LaBonte Lake, Laramie, Wyoming, sample from peak-bloom 1 (Peak bloom metagenome 1) | Fresh water microbial communities from LaBonte Lake | 1488 | 2237 | 2290 | 725 | 33499501 | 0.226 |
| 2189573023 | Fresh water microbial communities from LaBonte Lake, Laramie, Wyoming, sample from algal/cyanobacterial bloom material peak-bloom 2 (algal/cyano bloom peak-bloom 2) | Fresh water microbial communities from LaBonte Lake | 1605 | 2475 | 2542 | 780 | 36907156 | 0.222 |
| 2166559025 | Line P sample_A_09_P04_1300 (A_09_P04_1300 June 2011 assembly) | Marine microbial communities from the Eastern Subtropical North Pacific Ocean, Expanding Oxygen minimum zones | 1143 | 1963 | 1905 | 570 | 26774899 | 0.217 |
| 2189573006 | Line P sample_A_09_P04_500 (sample_A_09_P04_500 June 2011 assem) | Marine microbial communities from the Eastern Subtropical North Pacific Ocean, Expanding Oxygen minimum zones | 1088 | 1919 | 1873 | 539 | 27748673 | 0.218 |
| 2189573007 | Line P sample_A_09_P04_1000 (A_09_P04_1000 June 2011 assem) | Marine microbial communities from the Eastern Subtropical North Pacific Ocean, Expanding Oxygen minimum zones | 882 | 1546 | 1461 | 458 | 18559048 | 0.221 |
| 2189573008 | Line P sample_F_10_SI03_200 (sample_F_10_SI03_200 June 2011 assem) | Marine microbial communities from the Eastern Subtropical North Pacific Ocean, Expanding Oxygen minimum zones | 1281 | 1901 | 1900 | 618 | 28483156 | 0.227 |
| 2189573009 | Line P sample_A_09_P04_10 (sample_A_09_P04_10 June 2011 assem) | Marine microbial communities from the Eastern Subtropical North Pacific Ocean, Expanding Oxygen minimum zones | 1283 | 2137 | 2115 | 622 | 30552856 | 0.220 |
| 2189573010 | Line P sample_A_09_P20_1000 (sample_A_09_P20_1000 June 2011 assem) | Marine microbial communities from the Eastern Subtropical North Pacific Ocean, Expanding Oxygen minimum zones | 1137 | 1925 | 1870 | 568 | 25981127 | 0.218 |
| 2189573011 | Line P sample_A_09_P20_500 (sample_A_09_P20_500 June 2011 assem) | Marine microbial communities from the Eastern Subtropical North Pacific Ocean, Expanding Oxygen minimum zones | 1115 | 1904 | 1853 | 559 | 27720387 | 0.219 |
| 2189573012 | Line P sample_J_08_P26_500 (sample_J_08_P26_500 June 2011 assem) | Marine microbial communities from the Eastern Subtropical North Pacific Ocean, Expanding Oxygen minimum zones | 1144 | 1903 | 1860 | 563 | 26668688 | 0.222 |

**Table S1. IMG/M metagenomics samples used in our study. (continued)**

| Taxon ID | Sample Name | Metagenomics Dataset | # of Enz | # of Cpd | # of Rxn | # of Rxn by CPE | Running Time(ms) | Acceptance Rate |
|---|---|---|---|---|---|---|---|---|
| 2189573013 | Line P sample_J_09_P20_500 (sample_J_09_P20_500 June 2011 assem) | Marine microbial communities from the Eastern Subtropical North Pacific Ocean, Expanding Oxygen minimum zones | 1115 | 1888 | 1863 | 545 | 27959100 | 0.219 |
| 2189573014 | Line P sample_J_09_P20_1000 (sample_J_09_P20_1000 June 2011 assem) | Marine microbial communities from the Eastern Subtropical North Pacific Ocean, Expanding Oxygen minimum zones | 1155 | 1960 | 1917 | 571 | 28545449 | 0.220 |
| 2189573015 | Line P sample F_10_SI03_10 (sample_F_10_SI03_10 June 2011 assem) | Marine microbial communities from the Eastern Subtropical North Pacific Ocean, Expanding Oxygen minimum zones | 1396 | 2213 | 2204 | 671 | 31725053 | 0.222 |
| 2189573016 | Line P sample_F_10_SI03_100 (sample_F_10_SI03_100 June 2011 assem) | Marine microbial communities from the Eastern Subtropical North Pacific Ocean, Expanding Oxygen minimum zones | 1416 | 2230 | 2228 | 666 | 32929189 | 0.221 |
| 2189573017 | Line P sample_F_10_SI03_120 (sample_F_10_SI03_120 June 2011 assem) | Marine microbial communities from the Eastern Subtropical North Pacific Ocean, Expanding Oxygen minimum zones | 1304 | 2086 | 2083 | 638 | 30454437 | 0.220 |
| 2189573018 | Line P sample_F_10_SI03_135 (sample_F_10_SI03_135 June 2011 assem) | Marine microbial communities from the Eastern Subtropical North Pacific Ocean, Expanding Oxygen minimum zones | 1346 | 2125 | 2149 | 654 | 31967917 | 0.223 |
| 2189573019 | Line P sample_F_10_SI03_150 (sample_F_10_SI03_150 June 2011 assem) | Marine microbial communities from the Eastern Subtropical North Pacific Ocean, Expanding Oxygen minimum zones | 1330 | 2106 | 2109 | 638 | 29861482 | 0.221 |
| 2001200001 | Soil microbial communities from Minnesota Farm | Soil microbial communities from Waseca County, Minnesota Farm | 1332 | 2123 | 2124 | 650 | 32041804 | 0.224 |
| 2124908025 | Miscanthus rhizosphere microbial communities from Kellogg Biological Station, MSU, sample from Bulk Soil Replicate 2: eDNA_1 (Bulk soil 2 January 2011 combined assembly) | Soil microbial communities from Miscanthus in Kellogg Biological Station, MSU | 949 | 1700 | 1614 | 471 | 22519790 | 0.214 |
| 2124908027 | Miscanthus rhizosphere microbial communities from Kellogg Biological Station, MSU, sample Rhizosphere Soil Replicate 2: eDNA_1 (Rhizo 2 January 2011 combined assembly) | Soil microbial communities from Miscanthus in Kellogg Biological Station, MSU | 1134 | 1954 | 1914 | 568 | 26957509 | 0.214 |
| 2124908038 | Soil microbial communities from permafrost in Bonanza Creek, Alaska, sample from Bog Site B3 (B3_all_CLC) | Soil microbial communities from permafrost in Bonanza Creek, Alaska | 1519 | 2193 | 2270 | 737 | 34021839 | 0.228 |

**Table S1. IMG/M metagenomics samples used in our study. (continued)**

| Taxon ID | Sample Name | Metagenomics Dataset | # of Enz | # of Cpd | # of Rxn | # of Rxn by CPE | Running Time(ms) | Acceptance Rate |
|---|---|---|---|---|---|---|---|---|
| 2124908040 | Soil microbial communities from permafrost in Bonanza Creek, Alaska, sample from Bog Site B4 (B4_CLC) | Soil microbial communities from permafrost in Bonanza Creek, Alaska | 1411 | 2079 | 2131 | 680 | 31622930 | 0.227 |
| 2124908041 | Soil microbial communities from permafrost in Bonanza Creek, Alaska, sample from Permafrost Layer P3 (P3_CLC) | Soil microbial communities from permafrost in Bonanza Creek, Alaska | 1497 | 2249 | 2313 | 735 | 34875075 | 0.226 |
| 2124908043 | Soil microbial communities from permafrost in Bonanza Creek, Alaska, sample from Active Layer A2 (A2_CLC_pe) | Soil microbial communities from permafrost in Bonanza Creek, Alaska | 1394 | 2196 | 2250 | 688 | 34149252 | 0.227 |
| 2124908044 | Soil microbial communities from permafrost in Bonanza Creek, Alaska, sample from Active Layer A5 (A5_CLC_pe) | Soil microbial communities from permafrost in Bonanza Creek, Alaska | 1436 | 2189 | 2239 | 711 | 31900709 | 0.227 |
| 2140918006 | Soil microbial communities from permafrost in Bonanza Creek, Alaska, sample from Permafrost Layer P1 (P1_CLC_pe) | Soil microbial communities from permafrost in Bonanza Creek, Alaska | 1542 | 2238 | 2313 | 749 | 32561269 | 0.227 |
| 2162886011 | Miscanthus rhizosphere microbial communities from Kellogg Biological Station, MSU, sample Rhizosphere Soil Replicate 1: eDNA_1 (Rhizosphere replicate 1 April 2011 assembly) | Soil microbial communities from Miscanthus in Kellogg Biological Station, MSU | 1294 | 2081 | 2083 | 634 | 30553746 | 0.222 |
| 2162886012 | Miscanthus rhizosphere microbial communities from Kellogg Biological Station, MSU, sample Bulk Soil Replicate 1 : eDNA_1 (Bulk soil 1 April 2011 assembly) | Soil microbial communities from Miscanthus in Kellogg Biological Station, MSU | 1352 | 2145 | 2188 | 666 | 32410244 | 0.225 |
