# Supplementary material for: Probabilistic Inference of Biochemical Reactions in Microbial Communities from Metagenomic Sequences
Source: PLoS Comput Biol. 2013 Mar 21;9(3):e1002981. doi: 10.1371/journal.pcbi.1002981 (PMC3605055; doi:10.1371/journal.pcbi.1002981)
Supplement: Table S2 — Reactions in Antarctic deep lake and Alaska permafrost samples. The first p-value is from the Fisher's test that checks if the occurrences of the reactions in the two groups of samples are different. This p-value equaling 1 means the reaction occurs in all the samples in the two groups. The second p-value is the t-test based on the marginal probability of the reactions. The last two columns are the average marginal probabilities of the reactions in the two groups of samples. (PDF) [file pcbi.1002981.s003.pdf]

Table S2. Reactions in Antarctic deep lake and Alaska permafrost samples. The first p-value is from the Fisher's test that checks if the occurrences of the reactions in the two groups of samples are different. This p-value equaling 1 means the reaction occurs in all the samples in the two groups. The second p-value is the t-test based on the marginal probability of the reactions. The last two columns are the average marginal probabilities of the reactions in the two groups of samples.

Kegg Rxn	Definition	p-value (Fisher's test, r=1 or 0)	p-value (t-test, $P(r\|\mathcal{M})$)	Avg. $P(r\|\mathcal{M})$ (Antarctic Deep Lake)	Avg. $P(r\|\mathcal{M})$ (Alaska Permafrost)
R00014	Pyruvate + Thiamin diphosphate <=>2-(alpha-Hydroxyethyl)thiamine diphosphate + CO2	1.0000	0.0392	0.6419	0.7697
R00022	Chitobiose + H2O <=>2 N-Acetyl-D-glucosamine	1.0000	0.0271	0.4258	0.1183
R00024	D-Ribulose 1,5-bisphosphate + CO2 + H2O <=>2 3-Phospho-D-glycerate	1.0000	0.0285	0.6642	0.6690
R00032	beta-Carotene + Oxygen <=>2 Retinal	0.0022	0.0000	1.0000	0.0000
R00056	Dinucleotide + H2O <=>2 Mononucleotide	0.0606	0.0251	0.0320	0.0000
R00126	ADP + ATP <=>Orthophosphate + P1,P4-Bis(5'-adenosyl) tetraphosphate	0.0606	0.0250	0.4465	0.0000
R00127	ATP + AMP <=>2 ADP	1.0000	0.0230	0.8238	0.6986
R00148	NH3 + Oxygen + Ubiquinol <=>Hydroxylamine + H2O + Ubiquinone	0.0152	0.0041	0.0000	0.8333
R00149	2 ATP + NH3 + CO2 + H2O <=>2 ADP + Orthophosphate + Carbamoyl phosphate	0.0606	0.0250	0.0000	0.6667
R00160	FAD + H2O <=>AMP + FMN	0.0606	0.0250	0.3326	0.0000
R00174	ATP + Pyridoxal <=>ADP + Pyridoxal phosphate	0.0801	0.0176	0.1056	0.5311
R00184	P1,P4-Bis(5'-adenosyl) tetraphosphate + H2O <=>ATP + AMP	0.0606	0.0440	0.7131	0.2637
R00207	Pyruvate + Orthophosphate + Oxygen <=>Acetyl phosphate + H2O2 + CO2	0.0152	0.0041	0.1667	1.0000
R00215	(R)-Malate + NAD+ <=>Pyruvate + CO2 + NADH + H+	1.0000	0.0237	0.3205	0.1870
R00217	Oxaloacetate <=>Pyruvate + CO2	1.0000	0.0284	0.7954	0.7632
R00287	UDP-glucose + H2O <=>UMP + D-Glucose 1-phosphate	0.0606	0.0250	0.3345	0.0000
R00291	UDP-glucose <=>UDP-D-galactose	1.0000	0.0250	0.6499	0.8934
R00308	1,3-beta-D-Glucan + H2O <=>D-Glucose + 1,3-beta-D-Glucan	0.0606	0.0250	0.0000	0.6667
R00339	(R,R)-Tartaric acid <=>Oxaloacetate + H2O	0.0606	0.0250	0.3333	1.0000
R00353	Malonyl-CoA + Pyruvate <=>Acetyl-CoA + Oxaloacetate	0.0606	0.0239	0.2214	0.6700
R00369	L-Alanine + Glyoxylate <=>Pyruvate + Glycine	0.0152	0.0038	0.1099	0.6684
R00372	Glycine + 2-Oxoglutarate <=>Glyoxylate + L-Glutamate	0.0152	0.0041	0.1107	0.6633
R00375	dATP + DNA <=>Diphosphate + DNA	1.0000	0.0363	0.4667	0.4762
R00405	ATP + Succinate + CoA <=>ADP + Orthophosphate + Succinyl-CoA	1.0000	0.0337	0.9566	0.9542
R00416	UTP + N-Acetyl-alpha-D-glucosamine 1-phosphate <=>Diphosphate + UDP-N-acetyl-D-glucosamine	1.0000	0.0041	0.7216	1.0000
R00420	UDP-N-acetyl-D-glucosamine <=>UDP-N-acetyl-D-mannosamine	1.0000	0.0132	0.6710	0.6647
R00448	L-Lysine + Oxygen + NADPH + H+ <=>N6-Hydroxy-L-lysine + NADP+ + H2O	0.0152	0.0041	0.8333	0.0000
R00469	(-)-Ureidoglycolate + H2O <=>Glyoxylate + 2 NH3 + CO2	0.0606	0.0250	0.6667	0.0000
R00471	D-4-Hydroxy-2-oxoglutarate <=>Pyruvate + Glyoxylate	1.0000	0.0341	0.4103	0.4724
R00483	ATP + L-Aspartate + NH3 <=>AMP + Diphosphate + L-Asparagine	0.0152	0.0041	0.1667	1.0000
R00494	Glutathione + H2O <=>Cys-Gly + L-Glutamate	1.0000	0.0488	0.7088	0.6558
R00502	UTP + alpha-D-Galactose 1-phosphate <=>Diphosphate + UDP-D-galactose	0.0152	0.0041	0.1667	1.0000
R00507	3'-Phosphoadenylyl sulfate + H2O <=>Sulfate + Adenosine 3',5'-bisphosphate	0.0606	0.0250	0.3317	0.0000
R00522	Oxalate <=>Formate + CO2	0.0606	0.0250	0.0000	0.6667
R00554	ATP + L-Arginine <=>ADP + L-Arginine phosphate	0.0606	0.0250	0.3333	1.0000

Table S2. Reactions in Antarctic deep lake and Alaska permafrost samples. (continued)

Kegg Rxn	Definition	p-value (Fisher's test, r=1 or 0)	p-value (t-test, $P(r\|\mathcal{M})$)	Avg. $P(r\|\mathcal{M})$ (Antarctic Deep Lake)	Avg. $P(r\|\mathcal{M})$ (Alaska Permafrost)
R00616	ATP + Thiamin diphosphate <=>ADP + Thiamin triphosphate	0.0606	0.0204	0.0703	0.2690
R00619	ATP + Thiamine <=>AMP + Thiamin diphosphate	0.0606	0.0363	0.2977	0.8282
R00631	Aldehyde + NAD+ + H2O <=>Fatty acid + NADH + H+	1.0000	0.0362	0.1549	0.1675
R00661	Phosphoenolpyruvate <=>3-Phosphonopyruvate	0.0606	0.0250	0.3333	1.0000
R00669	N-Acetylornithine + H2O <=>Acetate + L-Ornithine	1.0000	0.0431	0.8351	0.7064
R00678	L-Tryptophan + Oxygen <=>L-Formylkynurenine	0.0606	0.0077	0.2568	1.0000
R00702	2 trans,trans-Farnesyl diphosphate <=>Diphosphate + Presqualene diphosphate	1.0000	0.0237	0.4512	0.2985
R00708	(S)-1-Pyrroline-5-carboxylate + NADP+ + 2 H2O <=>L-Glutamate + NADPH + H+	1.0000	0.0429	0.5359	0.5266
R00720	ITP + H2O <=>IMP + Diphosphate	1.0000	0.0456	0.3535	0.4407
R00722	ATP + IDP <=>ADP + ITP	1.0000	0.0467	0.3268	0.4292
R00731	L-Tyrosine + Oxygen <=>3,4-Dihydroxy-L-phenylalanine + H2O	0.0606	0.0330	0.0000	0.3156
R00747	Phosphonoacetaldehyde + H2O <=>Acetaldehyde + Orthophosphate	0.0152	0.0041	0.0000	0.8333
R00755	Acetaldehyde + Thiamin diphosphate <=>2-(alpha-Hydroxyethyl)thiamine diphosphate	0.0606	0.0230	0.2014	0.6139
R00782	L-Cysteine + H2O <=>Hydrogen sulfide + Pyruvate + NH3	1.0000	0.0433	0.6187	0.5970
R00816	Catechol + Oxygen <=>2-Hydroxymuconate semialdehyde	0.1818	0.0126	0.1590	0.4350
R00817	Catechol + Oxygen <=>cis,cis-Muconate	0.0606	0.0034	0.1026	0.4365
R00860	Sulfite + Acceptor + AMP <=>Adenylyl sulfate + Reduced acceptor	0.0606	0.0244	0.2115	0.6374
R00866	ATP + D-Fructose <=>ADP + D-Fructose 1-phosphate	0.0152	0.0041	0.7974	0.0000
R00889	GDP-mannose <=>GDP-L-galactose	0.0801	0.0182	0.0877	0.4365
R00890	GDP-mannose + 1,4-beta-D-Mannan <=>GDP + 1,4-beta-D-Mannan	1.0000	0.0030	0.1737	0.5710
R00921	Propanoyl-CoA + Orthophosphate <=>Propanoyl phosphate + CoA	1.0000	0.0430	0.3420	0.4971
R00930	(S)-Methylmalonyl-CoA + Pyruvate <=>Propanoyl-CoA + Oxaloacetate	0.0606	0.0263	0.2238	0.6655
R00945	5,10-Methylenetetrahydrofolate + Glycine + H2O <=>Tetrahydrofolate + L-Serine	1.0000	0.0242	0.8189	0.6676
R00955	UDP-glucose + alpha-D-Galactose 1-phosphate <=>D-Glucose 1-phosphate + UDP-D-galactose	0.0152	0.0041	0.1667	1.0000
R00962	ITP + Cytidine <=>IDP + CMP	1.0000	0.0449	0.3251	0.4282
R00969	P1,P4-Bis(5'-uridyl) tetraphosphate + H2O <=>UTP + UMP	0.0606	0.0116	0.2127	0.0626
R00970	ITP + Uridine <=>IDP + UMP	1.0000	0.0418	0.3249	0.4298
R00997	1-Aminocyclopropane-1-carboxylate + H2O <=>2-Oxobutanoate + NH3	0.0152	0.0041	0.1667	1.0000
R01026	Acetylcholine + H2O <=>Choline + Acetate	0.0606	0.0250	0.0000	0.6667
R01055	Uracil + D-Ribose 5-phosphate <=>Pseudouridine 5'-phosphate + H2O	0.0606	0.0250	0.3333	1.0000
R01063	D-Glyceraldehyde 3-phosphate + Orthophosphate + NADP+ <=>3-Phospho-D-glyceroyl phosphate + NADPH + H+	0.4545	0.0223	0.7482	0.3322
R01126	IMP + H2O <=>Inosine + Orthophosphate	1.0000	0.0263	0.4745	0.4510
R01127	IMP + H2O <=>1-(5'-Phosphoribosyl)-5-formamido-4-imidazolecarboxamide	1.0000	0.0250	1.0000	0.7786
R01132	IMP + Diphosphate <=>Hypoxanthine + 5-Phospho-alpha-D-ribose 1-diphosphate	1.0000	0.0488	0.5040	0.4994
R01137	ATP + dADP <=>ADP + dATP	1.0000	0.0259	0.4041	0.4247
R01138	dATP + Pyruvate <=>dADP + Phosphoenolpyruvate	1.0000	0.0287	0.4329	0.4547
R01142	Methane + Oxygen + NADH + H+ <=>Methanol + NAD+ + H2O	0.0152	0.0041	0.1667	1.0000
R01155	Putrescine + 2-Oxoglutarate <=>4-Aminobutyraldehyde + L-Glutamate	0.0606	0.0250	0.3333	1.0000
R01171	Butanoyl-CoA + NAD+ <=>Crotonoyl-CoA + NADH + H+	1.0000	0.0349	0.5623	0.4990

Table S2. Reactions in Antarctic deep lake and Alaska permafrost samples. (continued)

Kegg Rxn	Definition	p-value (Fisher's test, r=1 or 0)	p-value (t-test, $P(r\|\mathcal{M})$)	Avg. $P(r\|\mathcal{M})$ (Antarctic Deep Lake)	Avg. $P(r\|\mathcal{M})$ (Alaska Permafrost)
R01174	Butanoyl-CoA + Orthophosphate <=>CoA + Butanoylphosphate	0.0152	0.0041	0.1667	1.0000
R01185	Inositol 1-phosphate + H2O <=>myo-Inositol + Orthophosphate	1.0000	0.0047	0.1942	0.2838
R01186	myo-Inositol 4-phosphate + H2O <=>myo-Inositol + Orthophosphate	1.0000	0.0201	0.1954	0.1871
R01187	1D-myo-Inositol 3-phosphate + H2O <=>myo-Inositol + Orthophosphate	1.0000	0.0042	0.8157	0.7815
R01232	P1,P4-Bis(5'-guanosyl) tetraphosphate + H2O <=>GTP + GMP	0.0606	0.0115	0.2109	0.0620
R01287	O-Acetyl-L-homoserine + Hydrogen sulfide <=>L-Homocysteine + Acetate	1.0000	0.0063	0.6434	0.6635
R01300	ATP + 4-Hydroxybenzoate + CoA <=>AMP + Diphosphate + 4-Hydroxybenzoyl-CoA	0.0152	0.0041	0.1667	1.0000
R01312	Phosphatidylcholine + H2O <=>1,2-Diacyl-sn-glycerol + Choline phosphate	0.0022	0.0038	0.0000	0.3662
R01332	1,4-beta-D-Mannan + (n-1) H2O <=>n D-Mannose	0.0152	0.0041	0.0000	0.8333
R01353	ATP + Propanoate <=>ADP + Propanoyl phosphate	0.4545	0.0181	0.2215	0.6916
R01366	Acetoacetate <=>Acetone + CO2	0.0801	0.0179	0.1667	0.8333
R01401	5'-Methylthioadenosine + H2O <=>Adenine + 5-Methylthio-D-ribose	0.0606	0.0150	0.2598	0.8573
R01411	5-Methylcytosine + H2O <=>Thymine + NH3	1.0000	0.0401	0.1102	0.0959
R01419	Benzaldehyde + NAD+ + H2O <=>Benzoate + NADH + H+	0.1818	0.0327	0.1873	0.4319
R01459	Cholesterol + Oxygen <=>Cholest-4-en-3-one + H2O2	0.0606	0.0250	0.3333	1.0000
R01468	ATP + Ethanolamine <=>ADP + Ethanolamine phosphate	0.0152	0.0041	0.1667	1.0000
R01478	H2O + beta-D-Glucuronoside <=>D-Glucuronate + Alcohol	0.0606	0.0249	0.0610	0.1832
R01497	UDP-glucose + N-Acylsphingosine <=>UDP + Glucosylceramide	0.0801	0.0179	0.1667	0.8333
R01498	Glucosylceramide + H2O <=>D-Glucose + N-Acylsphingosine	0.0152	0.0041	0.1667	1.0000
R01505	(2S,3S)-2,3-Dihydro-2,3-dihydroxybenzoate + NAD+ <=>2,3-Dihydroxybenzoate + NADH + H+	0.0606	0.0250	0.0000	0.6667
R01516	2,3-Bisphospho-D-glycerate + H2O <=>3-Phospho-D-glycerate + Orthophosphate	0.0022	0.0052	0.2429	0.0000
R01526	ATP + D-Ribulose <=>ADP + D-Ribulose 5-phosphate	0.1818	0.0198	0.3660	0.9451
R01547	ATP + dAMP <=>ADP + dADP	1.0000	0.0237	0.3521	0.6032
R01548	dATP + Cytidine <=>dADP + CMP	1.0000	0.0454	0.4054	0.4266
R01549	dATP + Uridine <=>dADP + UMP	1.0000	0.0308	0.4058	0.4273
R01555	Maltose + Orthophosphate <=>D-Glucose + beta-D-Glucose 1-phosphate	0.0606	0.0250	0.3333	1.0000
R01560	Adenosine + H2O <=>Inosine + NH3	1.0000	0.0237	0.8543	0.7519
R01569	dTMP + H2O <=>Thymidine + Orthophosphate	1.0000	0.0439	0.3304	0.3238
R01602	alpha-D-Glucose <=>beta-D-Glucose	0.0606	0.0250	0.3333	1.0000
R01618	Adenylyl sulfate + ATP <=>Sulfate + P1,P4-Bis(5'-adenosyl) tetraphosphate	0.0606	0.0250	0.4420	0.0000
R01641	Sedoheptulose 7-phosphate + D-Glyceraldehyde 3-phosphate <=>D-Ribose 5-phosphate + D-Xylulose 5-phosphate	1.0000	0.0323	0.5682	0.5638
R01662	3-Phospho-D-glyceroyl phosphate <=>2,3-Bisphospho-D-glycerate	0.0022	0.0052	0.2445	0.0000
R01682	L-Cysteate <=>Taurine + CO2	1.0000	0.0295	0.8033	0.4514
R01688	ATP + Butanoic acid <=>ADP + Butanoylphosphate	0.0801	0.0179	0.1667	0.8333
R01698	Dihydrolipoamide + NAD+ <=>Lipoamide + NADH + H+	1.0000	0.0365	0.0793	0.0827
R01699	Pyruvate + Enzyme N6-(lipoyl)lysine <=>[Dihydrolipoyllysine-residue acetyltransferase] S-acetyldihydrolipoyllysine + CO2	1.0000	0.0359	0.5709	0.5204
R01708	Pyridoxine + NADP+ <=>Pyridoxal + NADPH + H+	0.0606	0.0250	0.3333	1.0000
R01711	Pyridoxine + Oxygen <=>Pyridoxal + H2O2	0.4545	0.0312	0.2567	0.6022

Table S2. Reactions in Antarctic deep lake and Alaska permafrost samples. (continued)

Kegg Rxn	Definition	p-value (Fisher's test, r=1 or 0)	p-value (t-test, $P(r\|\mathcal{M})$)	Avg. $P(r\|\mathcal{M})$ (Antarctic Deep Lake)	Avg. $P(r\|\mathcal{M})$ (Alaska Permafrost)
R01751	(R,R)-Tartaric acid <=>D-Glycerate + CO2	1.0000	0.0239	0.5913	0.7864
R01770	Inosine + H2O <=>Hypoxanthine + D-Ribose	1.0000	0.0194	0.5398	0.4961
R01773	L-Homoserine + NAD+ <=>L-Aspartate 4-semialdehyde + NADH + H+	1.0000	0.0140	0.6705	0.6645
R01785	L-Xylulose 1-phosphate <=>Glycerone phosphate + Glycolaldehyde	0.0801	0.0168	0.0906	0.4620
R01788	alpha-D-Glucose 6-phosphate + H2O <=>alpha-D-Glucose + Orthophosphate	0.0022	0.0000	1.0000	0.0000
R01795	Tetrahydrobiopterin + L-Phenylalanine + Oxygen <=>Dihydrobiopterin + L-Tyrosine + H2O	0.0606	0.0250	0.3333	1.0000
R01802	CDP-diacylglycerol + myo-Inositol <=>CMP + 1-Phosphatidyl-D-myo-inositol	0.0606	0.0250	0.0000	0.6667
R01805	N-Acetylneuraminate 9-phosphate + H2O <=>N-Acetylneuraminate + Orthophosphate	0.0801	0.0179	0.1667	0.8333
R01825	D-Erythrose 4-phosphate + NAD+ + H2O <=>4-Phospho-D-erythronate + NADH + H+	0.0152	0.0041	0.8333	0.0000
R01829	Sedoheptulose 1,7-bisphosphate <=>Glycerone phosphate + D-Erythrose 4-phosphate	1.0000	0.0033	0.1410	0.2139
R01845	Sedoheptulose 1,7-bisphosphate + H2O <=>Sedoheptulose 7-phosphate + Orthophosphate	0.0152	0.0041	0.0000	0.1713
R01874	D-Cysteine + H2O <=>Hydrogen sulfide + NH3 + Pyruvate	0.0606	0.0250	0.3333	1.0000
R01895	Ribitol + NAD+ <=>D-Ribulose + NADH + H+	0.0801	0.0179	0.1667	0.8333
R01902	ATP + L-Xylulose <=>ADP + L-Xylulose 1-phosphate	0.0152	0.0190	0.1225	0.5390
R01906	L-Xylulose <=>L-Lyxose	0.0606	0.0077	0.1900	0.7040
R01909	ATP + Pyridoxine <=>ADP + Pyridoxine phosphate	0.0801	0.0174	0.1053	0.5311
R01942	S-Adenosyl-L-methionine + Caffeoyl-CoA <=>S-Adenosyl-L-homocysteine + Feruloyl-CoA	0.0606	0.0358	0.2352	0.6605
R01978	(S)-3-Hydroxy-3-methylglutaryl-CoA + CoA <=>Acetyl-CoA + H2O + Acetoacetyl-CoA	0.0022	0.0000	1.0000	0.0000
R01982	Pectate + H2O <=>D-Galacturonate + Pectate	0.0152	0.0059	0.0000	0.6956
R01983	D-Galacturonate <=>D-Tagaturonate	0.1818	0.0005	0.1010	0.5908
R02027	Phosphatidylglycerol + H2O <=>1,2-Diacyl-sn-glycerol + sn-Glycerol 3-phosphate	0.0022	0.0000	0.0000	0.5741
R02052	Phosphatidylethanolamine + H2O <=>1,2-Diacyl-sn-glycerol + Ethanolamine phosphate	0.0022	0.0000	0.0000	0.5783
R02054	Phosphatidylethanolamine + H2O <=>2-Acyl-sn-glycero-3-phosphoethanolamine + Fatty acid	0.0801	0.0381	0.5907	0.1303
R02073	Diphosphate + beta-D-Fructose 6-phosphate <=>Orthophosphate + beta-D-Fructose 1,6-bisphosphate	0.0606	0.0250	0.3333	1.0000
R02078	3,4-Dihydroxy-L-phenylalanine + L-Tyrosine + Oxygen <=>Dopaquinone + 3,4-Dihydroxy-L-phenylalanine + H2O	0.0606	0.0284	0.0000	0.0836
R02088	dAMP + H2O <=>Deoxyadenosine + Orthophosphate	1.0000	0.0269	0.2341	0.5025
R02089	ATP + Deoxyadenosine <=>ADP + dAMP	0.0606	0.0250	0.3333	1.0000
R02094	ATP + dTMP <=>ADP + dTDP	1.0000	0.0228	0.5692	0.5650
R02109	Starch + H2O <=>Amylose + alpha-D-Glucose	0.0152	0.0042	0.0838	0.4995
R02194	ATP + Ferulate + CoA <=>AMP + Diphosphate + Feruloyl-CoA	1.0000	0.0428	0.1494	0.2857
R02221	ATP + Sinapate + CoA <=>AMP + Diphosphate + Sinapoyl-CoA	1.0000	0.0457	0.1140	0.1975
R02225	ATP + Streptomycin <=>ADP + Streptomycin 6-phosphate	0.0606	0.0250	0.0000	0.6667
R02240	ATP + 1,2-Diacyl-sn-glycerol <=>ADP + Phosphatidate	0.0606	0.0250	0.3333	1.0000
R02263	L-Rhamnulose 1-phosphate <=>Glycerone phosphate + (S)-Lactaldehyde	0.0801	0.0124	0.0869	0.4853

Table S2. Reactions in Antarctic deep lake and Alaska permafrost samples. (continued)

Kegg Rxn	Definition	p-value (Fisher's test, r=1 or 0)	p-value (t-test, $P(r\|\mathcal{M})$)	Avg. $P(r\|\mathcal{M})$ (Antarctic Deep Lake)	Avg. $P(r\|\mathcal{M})$ (Alaska Permafrost)
R02287	5-Formiminotetrahydrofolate + L-Glutamate <=>Tetrahydrofolate + N-Formimino-L-glutamate	0.0606	0.0167	0.2212	0.7052
R02289	5-Methyltetrahydrofolate + Corrinoid + H+ <=>Methylcorrinoid + Tetrahydrofolate	0.0606	0.0250	0.0000	0.6667
R02361	Pectate <=>4-(4-Deoxy-alpha-D-gluc-4-enuronosyl)-D-galacturonate + Pectate	0.0606	0.0250	0.0000	0.5956
R02363	2 p-Benzenediol + Oxygen <=>2 p-Benzoquinone + 2 H2O	0.0606	0.0273	0.0000	0.2682
R02376	Propane-1,2-diol <=>Propanal + H2O	0.0801	0.0179	0.1667	0.8333
R02383	Tyramine + Oxygen + NADH + H+ <=>Dopamine + NAD+ + H2O	0.0606	0.0355	0.0000	0.1865
R02422	Allantoate + H2O <=>(-)-Ureidoglycolate + Urea	0.0152	0.0041	0.0000	0.8333
R02426	CDP-glucose <=>CDP-4-dehydro-6-deoxy-D-glucose + H2O	0.0152	0.0041	0.1667	1.0000
R02433	L-Cysteate + 2-Oxoglutarate <=>3-Sulfopyruvate + L-Glutamate	1.0000	0.0261	0.4345	0.2942
R02437	L-Rhamnose <=>L-Rhamnulose	0.0606	0.0250	0.3333	1.0000
R02451	S-Benzoate coenzyme A + Reduced acceptor + 2 ATP + 2 H2O <=>S-1,5-Cyclohexadiene-1-carboxylate coenzymeA + Acceptor + 2 Orthophosphate + 2 ADP	0.0022	0.0000	0.0000	1.0000
R02464	Sphinganine 1-phosphate <=>Ethanolamine phosphate + Hexadecanal	0.0152	0.0040	0.0849	0.5105
R02476	Androsterone + NAD+ <=>5alpha-Androstane-3,17-dione + NADH + H+	0.0152	0.0070	0.0000	0.1210
R02477	Androsterone + NADP+ <=>5alpha-Androstane-3,17-dione + NADPH + H+	0.0152	0.0074	0.0000	0.1229
R02545	meso-Tartaric acid + NAD+ <=>2-Hydroxy-3-oxosuccinate + NADH + H+	1.0000	0.0209	0.1284	0.0814
R02555	D-Altronate + NAD+ <=>D-Tagaturonate + NADH + H+	0.0152	0.0041	0.1667	1.0000
R02556	Deoxyadenosine + H2O <=>Deoxyinosine + NH3	1.0000	0.0239	0.2361	0.3937
R02557	Deoxyadenosine + Orthophosphate <=>Adenine + 2-Deoxy-D-ribose 1-phosphate	0.4545	0.0358	0.2376	0.4960
R02577	Propane-1,2-diol + NADP+ <=>Lactaldehyde + NADPH + H+	1.0000	0.0225	0.1455	0.3412
R02602	gamma-Oxalocrotonate <=>2-Hydroxy-2,4-pentadienoate + CO2	0.1818	0.0276	0.3891	0.9358
R02637	3-Dehydro-L-gulonate + NAD+ <=>(4R,5S)-4,5,6-Trihydroxy-2,3-dioxohexanoate + NADH + H+	0.0152	0.0041	0.0000	0.5554
R02639	3-Dehydro-L-gulonate + NADP+ <=>(4R,5S)-4,5,6-Trihydroxy-2,3-dioxohexanoate + NADPH + H+	0.0152	0.0041	0.0000	0.5571
R02658	ATP + 2-Keto-D-gluconic acid <=>ADP + 6-Phospho-2-dehydro-D-gluconate	0.0801	0.0179	0.8333	0.1667
R02661	2-Methylpropanoyl-CoA + Acceptor <=>2-Methylprop-2-enoyl-CoA + Reduced acceptor	1.0000	0.0098	0.1674	0.4834
R02664	2-Phospho-D-glycerate + ATP <=>2,3-Bisphospho-D-glycerate + ADP	0.0152	0.0041	0.1667	1.0000
R02691	UDP-D-galactose + 1,2-Diacyl-sn-glycerol <=>UDP + 1,2-Diacyl-3-beta-D-galactosyl-sn-glycerol	0.0152	0.0041	0.0000	0.8333
R02704	Protein N(pi)-phospho-L-histidine + Mannitol <=>Protein histidine + D-Mannitol 1-phosphate	0.0801	0.0179	0.1667	0.8333
R02720	XTP + H2O <=>Xanthosine 5'-phosphate + Diphosphate	1.0000	0.0057	0.1562	0.1286
R02727	alpha,alpha-Trehalose + Orthophosphate <=>D-Glucose + beta-D-Glucose 1-phosphate	0.0606	0.0250	0.0000	0.6667
R02739	alpha-D-Glucose 6-phosphate <=>beta-D-Glucose 6-phosphate	1.0000	0.0037	0.5755	0.5107
R02740	alpha-D-Glucose 6-phosphate <=>beta-D-Fructose 6-phosphate	1.0000	0.0039	0.5684	0.6504
R02782	2,4,6/3,5-Pentahydroxycyclohexanone <=>3D-(3,5/4)-Trihydroxycyclohexane-1,2-dione + H2O	0.0606	0.0084	0.2365	0.8955
R02805	P1,P4-Bis(5'-xanthosyl) tetraphosphate + H2O <=>XTP + Xanthosine 5'-phosphate	0.0606	0.0109	0.1026	0.0299
R02825	5-Aminopentanoate + Lipoate <=>D-Proline + Dihydrolipoate	0.0152	0.0041	0.0000	0.8333

Table S2. Reactions in Antarctic deep lake and Alaska permafrost samples. (continued)

Kegg Rxn	Definition	p-value (Fisher's test, r=1 or 0)	p-value (t-test, $P(r\|\mathcal{M})$)	Avg. $P(r\|\mathcal{M})$ (Antarctic Deep Lake)	Avg. $P(r\|\mathcal{M})$ (Alaska Permafrost)
R02872	Presqualene diphosphate + NADPH + H+ <=>Diphosphate + Squalene + NADP+	1.0000	0.0222	0.2344	0.3008
R02886	Cellulose + H2O <=>Cellulose + Cellobiose	1.0000	0.0264	0.7743	0.8926
R02926	Melibiitol + H2O <=>D-Sorbitol + D-Galactose	1.0000	0.0164	0.1607	0.1559
R02946	(R,R)-Butane-2,3-diol + NAD+ <=>(R)-Acetoin + NADH + H+	0.0606	0.0267	0.0406	0.1202
R02947	2-Acetolactate <=>(R)-Acetoin + CO2	0.0606	0.0250	0.0000	0.4303
R02948	(S)-2-Acetolactate <=>(R)-Acetoin + CO2	0.0606	0.0250	0.0000	0.3797
R02978	Sphinganine + NADP+ <=>3-Dehydrosphinganine + NADPH + H+	0.0152	0.0041	0.0000	0.8333
R02984	dTDP-glucose <=>dTDP-galactose	1.0000	0.0264	0.4269	0.2147
R02988	3-Oxoadipate + NAD+ <=>2-Maleylacetate + NADH + H+	0.0606	0.0272	0.1874	0.5533
R02989	3-Oxoadipate + NADP+ <=>2-Maleylacetate + NADPH + H+	0.0606	0.0272	0.1854	0.5471
R03014	ATP + L-Rhamnulose <=>ADP + L-Rhamnulose 1-phosphate	0.0152	0.0001	0.0501	0.5794
R03015	Formylmethanofuran + H2O + Acceptor <=>CO2 + Methanofuran + Reduced acceptor	0.0152	0.0043	0.1121	0.6656
R03036	Dephospho-CoA + H2O <=>Pantetheine 4'-phosphate + AMP	0.0606	0.0250	0.3359	0.0000
R03050	2-Acetolactate + Thiamin diphosphate <=>2-(alpha-Hydroxyethyl)thiamine diphosphate + Pyruvate	1.0000	0.0087	0.3208	0.4067
R03051	2-Acetolactate + NADPH + H+ <=>2,3-Dihydroxy-3-methylbutanoate + NADP+	1.0000	0.0460	0.4282	0.4803
R03062	Cephalosporin C + H2O <=>Deacetylcephalosporin C + Acetate	0.0152	0.0041	0.1667	1.0000
R03106	Hydrogen cyanide + Mercaptopyruvate <=>Thiocyanate + Pyruvate	1.0000	0.0079	0.1299	0.2184
R03122	Cyclomaltodextrin + H2O <=>Maltodextrin	0.0152	0.0041	0.1667	1.0000
R03132	O-Acetyl-L-serine + Thiosulfate <=>S-Sulfo-L-cysteine + Acetate	1.0000	0.0063	0.1175	0.1123
R03136	Polyvinyl alcohol + Ferricytochrome c <=>Oxidized polyvinyl alcohol + Ferrocytochrome c + H+	0.0152	0.0041	0.1667	1.0000
R03146	Formate + 2 Ferricytochrome b1 <=>CO2 + 2 Ferrocytochrome b1 + 2 H+	0.0606	0.0250	0.3333	1.0000
R03172	(S)-2-Methylbutanoyl-CoA + Acceptor <=>2-Methylbut-2-enoyl-CoA + Reduced acceptor	1.0000	0.0154	0.1637	0.4361
R03239	ITP + D-Tagatose 6-phosphate <=>IDP + D-Tagatose 1,6-bisphosphate	1.0000	0.0344	0.3067	0.4288
R03241	ATP + L-Fuculose <=>ADP + L-Fuculose 1-phosphate	0.0022	0.0000	0.0000	1.0000
R03244	L-Ribulose 5-phosphate <=>L-Xylulose 5-phosphate	0.0801	0.0179	0.1667	0.8333
R03270	2-(alpha-Hydroxyethyl)thiamine diphosphate + Enzyme N6-(lipoyl)lysine <=>[Dihydrolipoyllysine-residue acetyltransferase] S-acetyldihydrolipoyllysine + Thiamin diphosphate	1.0000	0.0206	0.4866	0.5173
R03275	(3S)-3,6-Diaminohexanoate <=>(3S,5S)-3,5-Diaminohexanoate	0.0152	0.0041	0.0000	0.8333
R03298	2,3-Bisphospho-D-glycerate + ATP <=>Cyclic 2,3-bisphospho-D-glycerate + ADP + Orthophosphate	0.0152	0.0041	0.0000	0.8333
R03321	beta-D-Glucose 6-phosphate <=>beta-D-Fructose 6-phosphate	1.0000	0.0044	0.5709	0.6517
R03332	1-Phosphatidyl-D-myo-inositol + H2O <=>Inositol 1-phosphate + 1,2-Diacyl-sn-glycerol	0.0022	0.0039	0.0000	0.2028
R03355	beta-D-Galactosyl-1,4-beta-D-glucosylceramide + H2O <=>Glucosylceramide + D-Galactose	1.0000	0.0035	0.0603	0.1190
R03370	Octadecanoyl-[acyl-carrier protein] + Reduced acceptor + Oxygen <=>Oleoyl-[acyl-carrier protein] + Acceptor + 2 H2O	0.0606	0.0276	0.1552	0.4570

Table S2. Reactions in Antarctic deep lake and Alaska permafrost samples. (continued)

Kegg Rxn	Definition	p-value (Fisher's test, r=1 or 0)	p-value (t-test, $P(r\|\mathcal{M})$)	Avg. $P(r\|\mathcal{M})$ (Antarctic Deep Lake)	Avg. $P(r\|\mathcal{M})$ (Alaska Permafrost)
R03390	Formylmethanofuran + 5,6,7,8-Tetrahydromethanopterin <=>Methanofuran + 5-Formyl-5,6,7,8-tetrahydromethanopterin	0.0152	0.0041	0.1667	1.0000
R03544	Butanal + NADH + H+ <=>1-Butanol + NAD+	1.0000	0.0341	0.2516	0.2169
R03545	Butanal + NADPH + H+ <=>1-Butanol + NADP+	1.0000	0.0271	0.2516	0.2166
R03549	Gallate + Oxygen <=>2-Pyrone-4,6-dicarboxylate + H2O	1.0000	0.0042	0.1596	0.4259
R03550	Gallate + Oxygen <=>4-Carboxy-2-hydroxyhexa-2,4-dienedioate	0.0606	0.0008	0.0836	0.4652
R03601	O-Acetyl-L-serine + Selenide <=>L-Selenocysteine + Acetate	1.0000	0.0242	0.5219	0.5301
R03647	ATP + L-Aspartate + tRNA(Asn) <=>AMP + Diphosphate + L-Aspartyl-tRNA(Asn)	0.1818	0.0079	0.0739	0.2377
R03648	ATP + L-Asparagine + tRNA(Asn) <=>AMP + Diphosphate + L-Asparaginyl-tRNA(Asn)	0.0606	0.0250	0.3333	1.0000
R03819	ATP + Sedoheptulose <=>ADP + Sedoheptulose 1-phosphate	0.0152	0.0041	0.0738	0.0000
R03966	2-Hydroxymuconate <=>gamma-Oxalocrotonate	0.0152	0.0041	0.1667	1.0000
R04018	Galactosylceramide + N-Acetylneuraminate <=>GM4 + H2O	0.0606	0.0250	1.0000	0.3333
R04019	Digalactosylceramide + H2O <=>Galactosylceramide + D-Galactose	1.0000	0.0271	0.1596	0.1541
R04034	Phosphatidylserine + H2O <=>2-Acyl-sn-glycero-3-phosphoserine + Fatty acid	0.0801	0.0190	0.1823	0.0322
R04143	ATP + 5-Methylthio-D-ribose <=>ADP + S-Methyl-5-thio-D-ribose 1-phosphate	0.0606	0.0250	0.3333	1.0000
R04147	Isopenicillin N <=>Penicillin N	0.0152	0.0041	0.1667	1.0000
R04171	3-Hydroxy-L-kynurenine + 2-Oxoglutarate <=>4-(2-Amino-3-hydroxyphenyl)-2,4-dioxobutanoate + L-Glutamate	0.1818	0.0382	0.0789	0.2063
R04199	2,3,4,5-Tetrahydrodipicolinate + NADP+ <=>L-2,3-Dihydrodipicolinate + NADPH + H+	1.0000	0.0117	0.6643	0.6698
R04203	(2S,3S)-3-Hydroxy-2-methylbutanoyl-CoA + NAD+ <=>2-Methylacetoacetyl-CoA + NADH + H+	1.0000	0.0297	0.0661	0.0708
R04204	(2S,3S)-3-Hydroxy-2-methylbutanoyl-CoA <=>2-Methylbut-2-enoyl-CoA + H2O	1.0000	0.0199	0.0709	0.0954
R04212	L-Asparaginyl-tRNA(Asn) + L-Glutamate + Orthophosphate + ADP <=>L-Aspartyl-tRNA(Asn) + L-Glutamine + ATP + H2O	1.0000	0.0361	0.0980	0.3124
R04224	2-Methylprop-2-enoyl-CoA + H2O <=>(S)-3-Hydroxyisobutyryl-CoA	1.0000	0.0408	0.0629	0.1450
R04258	4-Chlorocatechol + Oxygen <=>3-Chloro-cis,cis-muconate	0.0606	0.0328	0.0786	0.2322
R04277	2-Pyrone-4,6-dicarboxylate + H2O <=>4-Carboxy-2-hydroxyhexa-2,4-dienedioate	0.0606	0.0250	0.3333	1.0000
R04280	3-O-Methylgallate + Oxygen <=>2-Pyrone-4,6-dicarboxylate + Methanol	0.0606	0.0041	0.0555	0.2308
R04309	3alpha-Hydroxy-5beta-androstan-17-one + NAD+ <=>5beta-Androstane-3,17-dione + NADH + H+	0.0152	0.0041	0.0000	0.0894
R04310	3alpha-Hydroxy-5beta-androstan-17-one + NADP+ <=>5beta-Androstane-3,17-dione + NADPH + H+	0.0152	0.0041	0.0000	0.0881
R04347	5-Methyl-5,6,7,8-tetrahydromethanopterin + 2-Mercaptoethanesulfonate <=>5,6,7,8-Tetrahydromethanopterin + 2-(Methylthio)ethanesulfonate	0.0152	0.0041	0.0000	0.8333
R04371	Homoisocitrate <=>(Z)-But-1-ene-1,2,4-tricarboxylate + H2O	0.2424	0.0429	0.0351	0.2863
R04377	3-D-Glucosyl-1,2-diacylglycerol + UDP-glucose <=>Diglucosyl-diacylglycerol + UDP	1.0000	0.0394	0.4179	0.1443
R04382	4-(4-Deoxy-alpha-D-gluc-4-enuronosyl)-D-galacturonate <=>2 5-Dehydro-4-deoxy-D-glucuronate	0.0801	0.0179	0.1667	0.8333

Table S2. Reactions in Antarctic deep lake and Alaska permafrost samples. (continued)

Kegg Rxn	Definition	p-value (Fisher's test, r=1 or 0)	p-value (t-test, $P(r\|\mathcal{M})$)	Avg. $P(r\|\mathcal{M})$ (Antarctic Deep Lake)	Avg. $P(r\|\mathcal{M})$ (Alaska Permafrost)
R04440	(R)-2,3-Dihydroxy-3-methylbutanoate + NADP+ <=>3-Hydroxy-3-methyl-2-oxobutanoic acid + NADPH + H+	1.0000	0.0182	0.2370	0.2166
R04470	Digalactosyl-diacylglycerol + H2O <=>1,2-Diacyl-3-beta-D-galactosyl-sn-glycerol + D-Galactose	1.0000	0.0050	0.0646	0.1379
R04560	10-Formyltetrahydrofolate + 1-(5'-Phosphoribosyl)-5-amino-4-imidazolecarboxamide <=>Tetrahydrofolate + 1-(5'-Phosphoribosyl)-5-formamido-4-imidazolecarboxamide	1.0000	0.0205	0.4990	0.6122
R04672	(S)-2-Acetolactate + Thiamin diphosphate <=>2-(alpha-Hydroxyethyl)thiamine diphosphate + Pyruvate	1.0000	0.0124	0.2628	0.3601
R04693	(S)-N-Methylcoclaurine + Oxygen + Reduced acceptor <=>3'-Hydroxy-N-methyl-(S)-coclaurine + H2O + Acceptor	0.0606	0.0270	0.0000	0.0495
R04725	Dodecanoyl-[acyl-carrier protein] + NADP+ <=>trans-Dodec-2-enoyl-[acp] + NADPH + H+	0.0606	0.0294	0.0894	0.2607
R04781	4-Carboxy-4-hydroxy-2-oxoadipate <=>4-Carboxy-2-oxo-3-hexenedioate + H2O	0.5455	0.0291	0.1087	0.3293
R04786	Phytoene + Acceptor <=>Phytofluene + Reduced acceptor	1.0000	0.0175	0.4834	0.2373
R04787	Phytofluene + Acceptor <=>zeta-Carotene + Reduced acceptor	1.0000	0.0215	0.1924	0.1202
R04800	Neurosporene + Reduced acceptor + Oxygen <=>Lycopene + Acceptor + 2 H2O	1.0000	0.0166	0.3655	0.6773
R04803	beta-Zeacarotene + Oxygen + Reduced acceptor <=>gamma-Carotene + 2 H2O + Acceptor	0.0152	0.0189	0.0000	0.0913
R04818	3alpha,7alpha-Dihydroxy-5beta-cholestane + NAD+ <=>7alpha-Hydroxy-5beta-cholestan-3-one + NADH + H+	0.0152	0.0041	0.0000	0.0869
R04819	3alpha,7alpha-Dihydroxy-5beta-cholestane + NADP+ <=>7alpha-Hydroxy-5beta-cholestan-3-one + NADPH + H+	0.0152	0.0041	0.0000	0.0870
R04824	3alpha,7alpha,12alpha-Trihydroxy-5beta-cholestane + NAD+ <=>7alpha,12alpha-Dihydroxy-5beta-cholestan-3-one + NADH + H+	0.0152	0.0041	0.0000	0.0878
R04825	3alpha,7alpha,12alpha-Trihydroxy-5beta-cholestane + NADP+ <=>7alpha,12alpha-Dihydroxy-5beta-cholestan-3-one + NADPH + H+	0.0152	0.0042	0.0000	0.0896
R04829	Tetrahydrocortisone + NAD+ <=>17alpha,21-Dihydroxy-5beta-pregnane-3,11,20-trione + NADH + H+	0.0152	0.0042	0.0000	0.1109
R04830	17alpha,21-Dihydroxy-5beta-pregnane-3,11,20-trione + H+ + NADPH <=>Tetrahydrocortisone + NADP+	0.0152	0.0041	0.0000	0.1102
R04832	Urocortisol + NAD+ <=>11beta,17alpha,21-Trihydroxy-5beta-pregnane-3,20-dione + NADH + H+	0.0152	0.0042	0.0000	0.1111
R04833	Urocortisol + NADP+ <=>11beta,17alpha,21-Trihydroxy-5beta-pregnane-3,20-dione + NADPH + H+	0.0152	0.0042	0.0000	0.1128
R04834	Cortol + NAD+ <=>Urocortisol + NADH + H+	1.0000	0.0181	0.2168	0.2268
R04835	3alpha,11beta,21-Trihydroxy-20-oxo-5beta-pregnan-18-al + NAD+ <=>11beta,21-Dihydroxy-3,20-oxo-5beta-pregnan-18-al + NADH + H+	0.0152	0.0042	0.0000	0.0866
R04836	3alpha,11beta,21-Trihydroxy-20-oxo-5beta-pregnan-18-al + NADP+ <=>11beta,21-Dihydroxy-3,20-oxo-5beta-pregnan-18-al + NADPH + H+	0.0152	0.0042	0.0000	0.0880
R04837	Tetrahydrocorticosterone + NAD+ <=>11beta,21-Dihydroxy-5beta-pregnane-3,20-dione + NADH + H+	0.0152	0.0055	0.0000	0.1007

Table S2. Reactions in Antarctic deep lake and Alaska permafrost samples. (continued)

Kegg Rxn	Definition	p-value (Fisher's test, r=1 or 0)	p-value (t-test, $P(r\|\mathcal{M})$)	Avg. $P(r\|\mathcal{M})$ (Antarctic Deep Lake)	Avg. $P(r\|\mathcal{M})$ (Alaska Permafrost)
R04838	Tetrahydrocorticosterone + NADP+ <=>11beta,21-Dihydroxy-5beta-pregnane-3,20-dione + NADPH + H+	0.0152	0.0053	0.0000	0.0993
R04842	3alpha,21-Dihydroxy-5beta-pregnane-11,20-dione + NAD+ <=>21-Hydroxy-5beta-pregnane-3,11,20-trione + NADH + H+	0.0152	0.0045	0.0000	0.1230
R04843	3alpha,21-Dihydroxy-5beta-pregnane-11,20-dione + NADP+ <=>21-Hydroxy-5beta-pregnane-3,11,20-trione + NADPH + H+	0.0152	0.0046	0.0000	0.1229
R04845	3alpha-Hydroxy-5beta-pregnane-20-one + NAD+ <=>5beta-Pregnane-3,20-dione + NADH + H+	0.0152	0.0041	0.0000	0.1103
R04846	3alpha-Hydroxy-5beta-pregnane-20-one + NADP+ <=>5beta-Pregnane-3,20-dione + NADPH + H+	0.0152	0.0042	0.0000	0.1120
R04859	O-Acetyl-L-serine + Thiosulfate + Thioredoxin + H+ <=>L-Cysteine + Sulfite + Thioredoxin disulfide + Acetate	1.0000	0.0056	0.7110	0.6754
R04880	3,4-Dihydroxyphenylethyleneglycol + NAD+ <=>3,4-Dihydroxymandelaldehyde + NADH + H+	1.0000	0.0390	0.0766	0.0932
R04884	2 5,6-Dihydroxyindole + Oxygen <=>2 Indole-5,6-quinone + 2 H2O	0.0606	0.0270	0.0000	0.0716
R04903	5-Hydroxyindoleacetaldehyde + NAD+ + H2O <=>5-Hydroxyindoleacetate + H+ + NADH	1.0000	0.0230	0.1339	0.2634
R04905	S-Adenosyl-L-methionine + 5-Hydroxyindoleacetate <=>S-Adenosyl-L-homocysteine + 5-Methoxyindoleacetate	0.0606	0.0133	0.1974	0.6749
R04906	5-Hydroxyindoleacetate <=>5-Hydroxyindoleacetylglycine	0.0152	0.0041	0.1667	1.0000
R04917	5-(2'-Formylethyl)-4,6-dihydroxypicolinate + NADP+ + H2O <=>5-(2'-Carboxyethyl)-4,6-dihydroxypicolinate + NADPH + H+	1.0000	0.0272	0.0925	0.1056
R04927	2 Ferricytochrome c + Selenite + AMP <=>2 Ferrocytochrome c + Adenylylselenate	0.0606	0.0270	0.0606	0.1790
R04935	(5-L-Glutamyl)-peptide + Se-Methyl-L-selenocysteine <=>Peptide + gamma-Glutamyl-Se-methylselenocysteine	1.0000	0.0172	0.0423	0.0389
R04949	Cyanoglycoside + H2O <=>Cyanohydrin + D-Glucose	1.0000	0.0254	0.0630	0.0595
R04956	Hexanoyl-[acp] + NADP+ <=>trans-Hex-2-enoyl-[acp] + NADPH + H+	0.0606	0.0266	0.0430	0.1270
R04959	Octanoyl-[acp] + NADP+ <=>trans-Oct-2-enoyl-[acp] + NADPH + H+	0.0606	0.0325	0.0843	0.2410
R04962	Decanoyl-[acp] + NADP+ <=>trans-Dec-2-enoyl-[acp] + NADPH + H+	0.0606	0.0263	0.0911	0.2710
R04969	Hexadecanoyl-[acp] + NAD+ <=>trans-Hexadec-2-enoyl-[acp] + NADH + H+	1.0000	0.0026	0.1500	0.2252
R04970	Hexadecanoyl-[acp] + NADP+ <=>trans-Hexadec-2-enoyl-[acp] + NADPH + H+	0.0606	0.0050	0.0559	0.2138
R04979	Bilirubin beta-diglucuronide + 2 H2O + 3 Reduced acceptor <=>D-Urobilinogen + 2 D-Glucuronate + 3 Acceptor	0.0606	0.0288	0.0627	0.1830
R05133	Arbutin 6-phosphate + H2O <=>p-Benzenediol + beta-D-Glucose 6-phosphate	0.0606	0.0250	0.3333	1.0000
R05140	D-Glucose + Levan <=>Sucrose + Levan	0.0022	0.0000	1.0000	0.0000
R05177	Precorrin 8X <=>Hydrogenobyrinate	1.0000	0.0281	0.5079	0.7235
R05185	16 ATP + Nitrogen + 8 Reduced ferredoxin + 8 H+ + 16 H2O <=>16 Orthophosphate + 16 ADP + 8 Oxidized ferredoxin + 2 NH3 + Hydrogen	0.0606	0.0247	0.3178	0.9561
R05220	Cob(I)yrinate a,c diamide + ATP <=>Adenosyl cobyrinate a,c diamide + Triphosphate	1.0000	0.0474	0.5313	0.5679
R05221	Adenosyl cobinamide + ATP <=>Adenosyl cobinamide phosphate + ADP	1.0000	0.0318	0.5734	0.5059

Table S2. Reactions in Antarctic deep lake and Alaska permafrost samples. (continued)

Kegg Rxn	Definition	p-value (Fisher's test, r=1 or 0)	p-value (t-test, $P(r\|\mathcal{M})$)	Avg. $P(r\|\mathcal{M})$ (Antarctic Deep Lake)	Avg. $P(r\|\mathcal{M})$ (Alaska Permafrost)
R05224	Hydrogenobyrinate + 2 L-Glutamine + 2 ATP + 2 H2O <=>Hydrogenobyrinate a,c diamide + 2 Orthophosphate + 2 L-Glutamate + 2 ADP	1.0000	0.0270	0.5328	0.6849
R05226	Adenosyl cobyrinate hexaamide + (R)-1-Aminopropan-2-ol <=>Adenosyl cobinamide + H2O	0.0606	0.0250	1.0000	0.3333
R05258	p-Benzoquinone + Nitrite + NADP+ + H2O <=>4-Nitrophenol + Oxygen + NADPH + H+	1.0000	0.0062	0.1532	0.2969
R05300	4-Methylmuconolactone <=>3-Methyl-cis,cis-hexadienedioate	1.0000	0.0000	0.1702	0.1183
R05332	Acetyl-CoA + alpha-D-Glucosamine 1-phosphate <=>CoA + N-Acetyl-alpha-D-glucosamine 1-phosphate	1.0000	0.0043	0.6385	0.4982
R05365	2-Hydroxy-6-oxo-(2'-aminophenyl)-hexa-2,4-dienoate + H2O <=>2-Hydroxy-2,4-pentadienoate + Anthranilate	1.0000	0.0127	0.5062	0.4554
R05367	trans-1,3-Dichloropropene + H2O <=>trans-3-Chloro-2-propene-1-ol + Hydrochloric acid	1.0000	0.0420	0.1673	0.1720
R05390	3-Chloro-cis,cis-muconate <=>Protoanemonin + Hydrochloric acid + CO2	1.0000	0.0001	0.2190	0.1643
R05453	Acetophenone + CO2 <=>Benzoyl acetate + H+	0.0606	0.0250	0.0000	0.6667
R05496	Acetylene + Reduced ferredoxin + 2 H+ + ATP + H2O <=>Ethylene + Oxidized ferredoxin + ADP + Orthophosphate	0.0606	0.0287	0.0304	0.0889
R05571	Galactitol 1-phosphate + NAD+ <=>D-Tagatose 6-phosphate + NADH + H+	0.0606	0.0250	0.0000	0.6667
R05572	Deisopropylatrazine + H2O <=>Deisopropylhydroxyatrazine + Hydrochloric acid	1.0000	0.0194	0.4726	0.2996
R05581	6-Hydroxycyclohex-1-enecarbonyl-CoA + NAD+ <=>6-Ketoxycyclohex-1-ene-1-carboxyl-CoA + NADH + H+	0.0606	0.0250	0.0000	0.6667
R05583	2,6-Dihydroxycyclohexane-1-carboxyl-CoA + Acceptor <=>6-Oxo-2-hydroxycyclohexane-1-carboxyl-CoA + Reduced acceptor	1.0000	0.0189	0.1602	0.2267
R05592	2-Ketocyclohexane-1-carboxyl-CoA + H2O <=>6-Carboxyhexanoyl-CoA	0.0022	0.0000	0.0000	1.0000
R05593	6-Oxo-2-hydroxycyclohexane-1-carboxyl-CoA + H2O <=>3-Hydroxypimeloyl-CoA	1.0000	0.0134	0.5400	0.7046
R05594	6-Ketoxycyclohex-1-ene-1-carboxyl-CoA + 2 H2O <=>3-Hydroxypimeloyl-CoA	0.0606	0.0250	0.0000	0.6667
R05597	S-1,5-Cyclohexadiene-1-carboxylate coenzymeA + H2O <=>6-Hydroxycyclohex-1-enecarbonyl-CoA	0.0606	0.0250	0.0000	0.6667
R05602	6-Hydroxycyclohex-1-enecarbonyl-CoA + H2O <=>2,6-Dihydroxycyclohexane-1-carboxyl-CoA	1.0000	0.0056	0.0731	0.1999
R05620	Cyclohexane-1-carboxylate + CoA + ATP <=>Cyclohexane-1-carboxyl-CoA + AMP + Diphosphate	0.0606	0.0250	0.3333	1.0000
R05624	H2O + Levan(m+n) <=>Levan(m) + Levan(n)	0.0801	0.0179	0.1667	0.8333
R05645	Sedoheptulose 7-phosphate <=>D-glycero-D-manno-Heptose 7-phosphate	0.0606	0.0250	0.3333	1.0000
R05661	2-Deoxy-5-keto-D-gluconic acid + ATP <=>2-Deoxy-5-keto-D-gluconic acid 6-phosphate + ADP	0.0606	0.0250	0.3333	1.0000
R05712	NH3 + 2 H2O + 6 Ferricytochrome c <=>Nitrite + 6 Ferrocytochrome c + 6 H+	0.0152	0.0041	0.0000	0.8333
R05775	UDP-glucose + Sulfite <=>UDP-6-sulfoquinovose + H2O	0.0606	0.0250	0.3333	1.0000
R05813	Cobalt-dihydro-precorrin 6 + 2 S-Adenosyl-L-methionine <=>Cobalt-precorrin 8 + 2 S-Adenosyl-L-homocysteine + CO2	0.1818	0.0495	0.9133	0.4213
R05814	Cobalt-precorrin 8 <=>Cobyrinate	1.0000	0.0245	0.6159	0.3707

Table S2. Reactions in Antarctic deep lake and Alaska permafrost samples. (continued)

Kegg Rxn	Definition	p-value (Fisher's test, r=1 or 0)	p-value (t-test, $P(r\|\mathcal{M})$)	Avg. $P(r\|\mathcal{M})$ (Antarctic Deep Lake)	Avg. $P(r\|\mathcal{M})$ (Alaska Permafrost)
R05815	Cobyrinate + 2 L-Glutamine + 2 ATP + 2 H2O <=>Cob(II)yrinate a,c diamide + 2 L-Glutamate + 2 ADP + 2 Orthophosphate	1.0000	0.0270	0.6033	0.4453
R05843	Phenanthrene + NADH + H+ + Oxygen <=>(+)-cis-3,4-Dihydrophenanthrene-3,4-diol + NAD+	0.0152	0.0041	0.8333	0.0000
R05961	H2O + Globotriaosylceramide <=>D-Galactose + Lactosylceramide	1.0000	0.0129	0.0706	0.0649
R05963	H2O + Globoside <=>N-Acetyl-D-galactosamine + Globotriaosylceramide	1.0000	0.0212	0.0919	0.0514
R06004	GM2 + H2O <=>GM3 + N-Acetyl-D-galactosamine	1.0000	0.0292	0.0807	0.0488
R06117	(+)-trans-Carveol + NAD+ <=>(+)-(S)-Carvone + NADH + H+	0.0606	0.0250	0.3333	1.0000
R06180	(R,R)-Tartaric acid + NAD+ <=>2-Hydroxy-3-oxosuccinate + NADH + H+	1.0000	0.0223	0.1851	0.2234
R06200	1,4-beta-D-Glucan(n+2) + H2O <=>1,4-beta-D-Glucan(n) + Cellobiose	1.0000	0.0263	0.3179	0.2149
R06223	2 trans,trans-Farnesyl diphosphate + NADPH + H+ <=>Squalene + 2 Diphosphate + NADP+	1.0000	0.0237	0.6088	0.7755
R06266	13(1)-Hydroxy-magnesium-protoporphyrin IX 13-monomethyl ester + NADPH + H+ + Oxygen <=>13(1)-Oxo-magnesium-protoporphyrin IX 13-monomethyl ester + NADP+ + 2 H2O	0.1818	0.0307	0.1952	0.4573
R06267	13(1)-Oxo-magnesium-protoporphyrin IX 13-monomethyl ester + NADPH + H+ + Oxygen <=>Divinylprotochlorophyllide + NADP+ + 2 H2O	0.1818	0.0067	0.1907	0.5802
R06269	13(1)-Hydroxy-magnesium-protoporphyrin IX 13-monomethyl ester + Acceptor <=>13(1)-Oxo-magnesium-protoporphyrin IX 13-monomethyl ester + Reduced acceptor	0.0152	0.0033	0.0728	0.4558
R06270	13(1)-Oxo-magnesium-protoporphyrin IX 13-monomethyl ester + Acceptor <=>Divinylprotochlorophyllide + Reduced acceptor	0.0152	0.0001	0.0492	0.5810
R06282	Protochlorophyllide + ATP <=>Chlorophyllide + ADP + Orthophosphate	0.0022	0.0000	1.0000	0.0000
R06286	Divinyl chlorophyllide a + NADP+ <=>Divinylprotochlorophyllide + NADPH + H+	0.4545	0.0000	0.0868	0.6556
R06367	Perillic acid + CoA + ATP <=>Perillyl-CoA + H2O + ADP + Orthophosphate	1.0000	0.0402	0.1969	0.1704
R06368	Perillic acid + CoA + ATP <=>Perillyl-CoA + H2O + AMP + Diphosphate	1.0000	0.0389	0.1972	0.1696
R06369	Perillyl-CoA + H2O <=>2-Hydroxy-4-isopropenylcyclohexane-1-carboxyl-CoA	1.0000	0.0174	0.0992	0.0844
R06370	2-Hydroxy-4-isopropenylcyclohexane-1-carboxyl-CoA + Acceptor <=>4-Isopropenyl-2-oxy-cyclohexanecarboxyl-CoA + Reduced acceptor	1.0000	0.0017	0.0876	0.0791
R06407	(Z)-2-Methyl-5-isopropylhexa-2,5-dienal + NAD+ + H2O <=>cis-2-Methyl-5-isopropylhexa-2,5-dienoic acid + NADH + H+	1.0000	0.0381	0.0599	0.0669
R06413	3-Hydroxy-2,6-dimethyl-5-methylene-heptanoyl-CoA + NAD+ <=>2,6-Dimethyl-5-methylene-3-oxo-heptanoyl-CoA + NADH + H+	1.0000	0.0004	0.1259	0.1113
R06414	2,6-Dimethyl-5-methylene-3-oxo-heptanoyl-CoA + CoA <=>3-Isopropylbut-3-enoyl-CoA + Propanoyl-CoA	1.0000	0.0064	0.1953	0.1607
R06415	3-Isopropylbut-3-enoyl-CoA + H2O <=>3-Isopropylbut-3-enoic acid + CoA	1.0000	0.0132	0.4903	0.3358
R06435	dTDP-4-oxo-2,6-dideoxy-L-mannose + S-Adenosyl-L-methionine <=>dTDP-3-methyl-4-oxo-2,6-dideoxy-L-glucose + S-Adenosyl-L-homocysteine + H+	0.0152	0.0041	0.0000	0.8333
R06515	(3R)-3-Isopropenyl-6-oxoheptanoate + CoA + ATP <=>(3R)-3-Isopropenyl-6-oxoheptanoyl-CoA + H2O + AMP + Diphosphate	1.0000	0.0432	0.1665	0.1457
R06516	Sphingosine 1-phosphate <=>Ethanolamine phosphate + Hexadecenal	0.0152	0.0040	0.0856	0.5142

Table S2. Reactions in Antarctic deep lake and Alaska permafrost samples. (continued)

Kegg Rxn	Definition	p-value (Fisher's test, r=1 or 0)	p-value (t-test, $P(r\|\mathcal{M})$)	Avg. $P(r\|\mathcal{M})$ (Antarctic Deep Lake)	Avg. $P(r\|\mathcal{M})$ (Alaska Permafrost)
R06519	Dihydroceramide + Reduced acceptor + Oxygen <=>N-Acylsphingosine + Acceptor + 2 H2O	0.0152	0.0041	0.1667	1.0000
R06558	Adenosyl cobinamide + GTP <=>Adenosyl cobinamide phosphate + GDP	1.0000	0.0411	0.5708	0.5059
R06562	2'-Hydroxyformononetin + NADPH + H+ <=>(-)-Vestitone + NADP+	0.0152	0.0041	0.0000	0.2208
R06563	2'-Hydroxybiochanin A + NADPH + H+ <=>Ferreirin + NADP+	0.0152	0.0042	0.0000	0.2205
R06578	5-Hydroxyferuloyl-CoA + S-Adenosyl-L-methionine <=>Sinapoyl-CoA + S-Adenosyl-L-homocysteine	0.0606	0.0130	0.1122	0.3832
R06731	Pseudoecgonine + ATP + CoA <=>Pseudoecgonyl-CoA + AMP	1.0000	0.0453	0.0897	0.0782
R06833	3,4,6-Trichlorocatechol + Oxygen <=>2,3,5-Trichloro-cis,cis-muconate	0.0606	0.0433	0.0340	0.0918
R06839	3-Chlorocatechol + Oxygen <=>2-Chloro-cis,cis-muconate	0.0606	0.0416	0.0502	0.1434
R06848	2,5-Dichloro-4-oxohex-2-enedioate + 2 NADH + 2 H+ <=>2-Chloro-3-oxoadipate + Hydrochloric acid + 2 NAD+	0.0606	0.0147	0.0233	0.0766
R06854	2-Hydroxy-1,4-benzoquinone <=>p-Benzoquinone + H2O	1.0000	0.0090	0.1173	0.1611
R06907	Naphthyl-2-hydroxymethyl-succinyl CoA <=>Naphthyl-2-oxomethyl-succinyl-CoA + 2 H+	1.0000	0.0086	0.1169	0.1077
R06913	2-Hydroxy-3-methylbenzalpyruvate + H2O <=>3-Methylsalicylaldehyde + Pyruvate	1.0000	0.0499	0.0649	0.0579
R06917	1-Hydroxymethylnaphthalene + NAD+ <=>1-Naphthaldehyde + NADH + H+	1.0000	0.0221	0.0659	0.0749
R06918	1-Naphthaldehyde + NAD+ + H2O <=>1-Naphthoic acid + NADH + H+	1.0000	0.0129	0.0596	0.0709
R06927	(2-Naphthyl)methanol + NAD+ <=>2-Naphthaldehyde + NADH + H+	1.0000	0.0252	0.0719	0.0802
R06928	2-Naphthaldehyde + NAD+ + H2O <=>2-Naphthoic acid + NADH + H+	1.0000	0.0101	0.0915	0.1053
R06961	alpha-Zeacarotene <=>delta-Carotene	0.0152	0.0050	0.0000	0.0643
R06978	L-2,4-Diaminobutanoate + Acetyl-CoA <=>N-gamma-Acetyldiaminobutyrate + CoA	0.0606	0.0250	0.6667	0.0000
R06989	cis,cis-Muconate <=>(S)-5-Oxo-2,5-dihydrofuran-2-acetate	1.0000	0.0001	0.2029	0.4837
R06990	(S)-5-Oxo-2,5-dihydrofuran-2-acetate <=>2-Oxo-2,3-dihydrofuran-5-acetate	0.0152	0.0041	0.1667	1.0000
R07084	Benzo[a]pyrene-7,8-diol + Glutathione <=>7,8-Dihydro-7-hydroxy-8-S-glutathionyl-benzo[a]pyrene + H2O	1.0000	0.0389	0.0757	0.0589
R07105	Chloral hydrate + NADH + H+ <=>Trichloroethanol + NAD+ + H2O	1.0000	0.0152	0.0737	0.0873
R07157	CO + H2O + Oxidized ferredoxin <=>CO2 + Reduced ferredoxin	0.0606	0.0250	0.0000	0.6667
R07160	3-Methyl-2-oxobutanoic acid + CoA + 2 Oxidized ferredoxin <=>2-Methylpropanoyl-CoA + CO2 + 2 Reduced ferredoxin + H+	0.1818	0.0245	0.1909	0.4752
R07244	S-Adenosyl-L-methionine + N,N-Dimethylglycine <=>S-Adenosyl-L-homocysteine + Betaine	0.0152	0.0041	0.8333	0.0000
R07245	Carbamoyl phosphate + N-Acetylornithine <=>Orthophosphate + N-Acetyl-L-citrulline	0.0801	0.0179	0.1667	0.8333
R07268	ATP + Cobinamide <=>Triphosphate + Adenosyl cobinamide	1.0000	0.0346	0.5341	0.4896
R07306	GTP + 3 H2O <=>2-Amino-5-formylamino-6-(5-phospho-D-ribosylamino)pyrimidin-4(3H)-one + 2 Orthophosphate	0.0152	0.0041	1.0000	0.1667
R07322	Squalene <=>Diploptene	0.0606	0.0265	0.1783	0.5290
R07323	Squalene + H2O <=>Diplopterol	0.0606	0.0235	0.1761	0.5347
R07381	O-1-Alk-1-enyl-2-acyl-sn-glycero-3-phosphoethanolamine + H2O <=>1-Alkenyl-2-acylglycerol + Ethanolamine phosphate	0.0022	0.0000	0.0000	0.0590
R07413	Digalacturonate + H2O <=>2 D-Galacturonate	0.0152	0.0135	0.0000	0.2546

Table S2. Reactions in Antarctic deep lake and Alaska permafrost samples. (continued)

Kegg Rxn	Definition	p-value (Fisher's test, r=1 or 0)	p-value (t-test, $P(r\|\mathcal{M})$)	Avg. $P(r\|\mathcal{M})$ (Antarctic Deep Lake)	Avg. $P(r\|\mathcal{M})$ (Alaska Permafrost)
R07476	(2R)-O-Phospho-3-sulfolactate <=>Sulfite + Phosphoenolpyruvate	0.0606	0.0250	0.3333	1.0000
R07511	9,9'-Di-cis-zeta-carotene <=>7,9,7',9'-Tetra-cis-lycopene	0.0152	0.0065	0.0000	0.1297
R07599	3-Methyl-2-oxobutanoic acid + Thiamin diphosphate <=>2-Methyl-1-hydroxypropyl-ThPP + CO2	1.0000	0.0428	0.3451	0.3286
R07613	LL-2,6-Diaminoheptanedioate + 2-Oxoglutarate <=>2,3,4,5-Tetrahydrodipicolinate + L-Glutamate + H2O	0.0152	0.0041	0.1667	1.0000
R07658	UDP-glucuronate + NAD+ <=>UDP-L-Ara4O + CO2 + NADH + H+	0.1818	0.0284	0.7784	0.3318
R07672	GDP-mannose <=>GDP-L-gulose	0.0801	0.0176	0.0873	0.4384
R07673	GDP-L-gulose <=>GDP-L-galactose	0.0801	0.0165	0.0330	0.1689
R07677	L-Ascorbate 6-phosphate + H2O <=>3-Dehydro-L-gulonate 6-phosphate	0.0152	0.0041	1.0000	0.1667
R07688	Anthracene-9,10-dihydrodiol <=>9,10-Dihydroxyanthracene + 2 H+	1.0000	0.0099	0.0855	0.0798
R07697	Phenylboronic acid + Oxygen <=>Phenol + Boric acid	1.0000	0.0467	0.0614	0.0785
R07747	2',7-Dihydroxy-4',5'-methylenedioxyisoflavone + NADPH + H+ <=>(+)-Sophorol + NADP+	0.0152	0.0041	0.0000	0.2362
R07751	2',7-Dihydroxy-4',5'-methylenedioxyisoflavone + NADPH + H+ <=>(-)-Sophorol + NADP+	0.0152	0.0041	0.0000	0.2362
R07762	Hexadecanoyl-[acp] + Malonyl-[acyl-carrier protein] <=>3-Oxostearoyl-[acp] + Acyl-carrier protein + CO2	1.0000	0.0074	0.1172	0.1684
R07768	Octanoyl-[acp] + 2 Sulfur + 2 S-Adenosyl-L-methionine <=>Lipoyl-[acp] + 2 L-Methionine + 2 5'-Deoxyadenosine	1.0000	0.0256	0.5183	0.5607
R07775	Cobalt-precorrin 7 + S-Adenosyl-L-methionine <=>Cobalt-precorrin 8 + S-Adenosyl-L-homocysteine + CO2	0.0606	0.0293	0.2621	0.0900
R07781	2-Bromomaleylacetate + NADH + H+ <=>2-Maleylacetate + NAD+ + Bromide	0.0606	0.0299	0.0182	0.0527
R07795	3-Sulfocatechol + Oxygen + H2O <=>2-Hydroxymuconate + Sulfite	0.1818	0.0281	0.0954	0.2467
R07808	G06780 + H2O <=>G13033 + Sulfate	0.1818	0.0343	0.3433	0.7926
R07809	G13033 + H2O <=>G08421 + N-Acetyl-D-galactosamine	1.0000	0.0261	0.4562	1.0000
R07810	G06780 + H2O <=>G08421 + N-Acetyl-D-galactosamine	1.0000	0.0098	0.2136	0.4192
R07816	G13038 + H2O <=>G13039 + N-Acetyl-D-glucosamine	0.0152	0.0041	0.1667	1.0000
R07818	G13040 + H2O <=>G09660 + D-Glucuronate	0.0606	0.0132	0.0724	0.2453
R07839	(E)-4-Oxobut-1-ene-1,2,4-tricarboxylate <=>4-Carboxy-2-hydroxyhexa-2,4-dienedioate	0.4545	0.0033	0.2415	0.7045
R07891	OPC6-CoA + Acetyl-CoA <=>CoA + 3-Oxo-OPC8-CoA	1.0000	0.0263	0.0527	0.0485
R07916	2 Geranylgeranyl diphosphate <=>Phytoene + 2 Diphosphate	1.0000	0.0431	0.6041	0.5247
R08035	2,4-Diamino-6-hydroxylaminotoluene <=>2,4,6-Triaminotoluene	0.0152	0.0041	0.1667	1.0000
R08058	5,6,7,8-Tetrahydromethanopterin + Formaldehyde <=>5,10-Methylenetetrahydromethanopterin + H2O	0.0152	0.0041	0.1667	1.0000
R08059	5,10-Methylenetetrahydromethanopterin + NADP+ <=>5,10-Methenyltetrahydromethanopterin + NADPH	0.0606	0.0250	0.0000	0.6667
R08060	Formylmethanofuran + H2O <=>Formate + Methanofuran	0.0152	0.0041	0.1112	0.6683
R08084	beta-Citronellol + NADP+ <=>(R)-(+)-Citronellal + NADPH + H+	1.0000	0.0350	0.1152	0.1715
R08085	(-)-Citronellol + NADP+ <=>(S)-(-)-Citronellal + NADPH + H+	1.0000	0.0288	0.1137	0.1726
R08086	Geranial + NAD+ + H2O <=>Geranic acid + NADH + H+	1.0000	0.0168	0.0726	0.0849

Table S2. Reactions in Antarctic deep lake and Alaska permafrost samples. (continued)

Kegg Rxn	Definition	p-value (Fisher's test, r=1 or 0)	p-value (t-test, $P(r \mid \mathcal{M})$)	Avg. $P(r \mid \mathcal{M})$ (Antarctic Deep Lake)	Avg. $P(r \mid \mathcal{M})$ (Alaska Permafrost)
R08087	(R)-(+)-Citronellal + NAD+ + H2O <=>Citronellate + NADH + H+	1.0000	0.0286	0.5048	0.4276
R08088	Citronellate + CoA + ATP <=>Citronellyl-CoA + AMP + Diphosphate	0.0606	0.0250	1.0000	0.3333
R08096	(S)-(-)-Citronellal + NAD+ + H2O <=>Citronellate + NADH + H+	1.0000	0.0313	0.4970	0.4302
R08105	4-Fluorocyclohexadiene-cis,cis-1,2-diol + NAD+ <=>4-Fluorocatechol + NADH + H+	0.1818	0.0402	0.0443	0.0988
R08114	3-Fluorocatechol + Oxygen <=>2-Fluoro-cis,cis-muconate	0.0606	0.0394	0.0349	0.0960
R08115	4-Fluorocatechol + Oxygen <=>3-Fluoro-cis,cis-muconate	0.0606	0.0370	0.0420	0.1165
R08116	3-Fluoro-cis,cis-muconate <=>4-Fluoromuconolactone	1.0000	0.0001	0.1704	0.1269
R08127	Luteolin 7-O-[beta-D-glucuronosyl-(1->2)-beta-D-glucuronide]-4'-O-beta-D-glucuronide + H2O <=>Luteolin 7-O-[beta-D-glucuronosyl-(1->2)-beta-D-glucuronide] + D-Glucuronate	0.0606	0.0278	0.0628	0.1845
R08161	Hexadecanoyl-[acp] + Reduced acceptor + Oxygen <=>Hexadecenoyl-[acyl-carrier protein] + Acceptor + 2 H2O	0.0606	0.0219	0.1883	0.5789
R08247	beta-D-Ribopyranose <=>beta-D-Ribofuranose	0.0152	0.0041	0.1667	1.0000
R08260	SN38 glucuronide + H2O <=>SN-38 + D-Glucuronate	0.0606	0.0318	0.1138	0.3265
R08366	N-Acetyl-D-galactosamine + Protein N(pi)-phospho-L-histidine <=>N-Acetyl-D-galactosamine 6-phosphate + Protein histidine	0.0152	0.0041	0.0000	0.8333
R08503	5-Deoxy glucuronic acid <=>2-Deoxy-5-keto-D-gluconic acid	0.0152	0.0041	0.1667	1.0000
R08553	AMP + Sulfite + FAD <=>Adenylyl sulfate + FADH2	0.0606	0.0257	0.2128	0.6353
R08555	N-Acetylmuramic acid 6-phosphate + H2O <=>N-Acetyl-D-glucosamine 6-phosphate + (R)-Lactate	0.0606	0.0250	0.3333	1.0000
R08567	(S)-3-Methyl-2-oxopentanoic acid + CoA + 2 Oxidized ferredoxin <=>(S)-2-Methylbutanoyl-CoA + CO2 + 2 Reduced ferredoxin + H+	0.1818	0.0265	0.1570	0.3854
R08576	O-Phospho-L-serine + tRNA(Cys) + ATP <=>O-Phosphoseryl-tRNA(Cys) + Diphosphate + AMP	0.0606	0.0250	0.0000	0.6667
R08586	Chorismate <=>Futalosine	0.0801	0.0179	0.1667	0.8333
R08587	Futalosine + H2O <=>de-Hypoxanthine futalosine + Hypoxanthine	0.0606	0.0250	0.0000	0.6667
R08588	de-Hypoxanthine futalosine <=>Cyclic de-hypoxanthine futalosine	0.0152	0.0041	0.0000	0.8333
R08937	Carbamoyl phosphate + N2-Succinyl-L-ornithine <=>Orthophosphate + N-Succinyl-L-citrulline	0.0801	0.0179	0.1667	0.8333
R08959	5alpha-Pregnane-3alpha,20alpha-diol + NADP+ <=>3alpha-Hydroxy-5alpha-pregnan-20-one + NADPH + H+	1.0000	0.0012	0.2185	0.1846
R08963	Dihydrotestosterone + NADPH + H+ <=>Androstan-3alpha,17beta-diol + NADP+	0.0152	0.0083	0.0000	0.0685
R09058	Bacteriochlorophyllide a + Geranylgeranyl diphosphate + 3 NADPH + 3 H+ <=>Bacterio-chlorophyll a + Diphosphate + 3 NADP+	0.2424	0.0463	0.2165	0.0174
R09096	Acetyl-CoA + H+ + 5,6,7,8-Tetrahydromethanopterin <=>5-Methyl-5,6,7,8-tetrahydromethanopterin + CO + CoA	0.0801	0.0123	0.0393	0.3669
R09099	L-Serine + 5,6,7,8-Tetrahydromethanopterin <=>5,10-Methylenetetrahydromethanopterin + Glycine + H2O	1.0000	0.0237	0.3638	0.6679
R09103	Bis(4-hydroxyphenyl)methanol <=>4,4'-Dihydroxybenzophenone	1.0000	0.0306	0.1090	0.1043
R09107	N-Acetyl-L-citrulline + H2O <=>Acetate + L-Citrulline	1.0000	0.0229	0.3060	0.5918
R09134	Tetrachlorocatechol + Oxygen <=>Tetrachloro-cis,cis-muconate	0.0606	0.0385	0.0523	0.1445

Table S2. Reactions in Antarctic deep lake and Alaska permafrost samples. (continued)

Kegg Rxn	Definition	p-value (Fisher's test, r=1 or 0)	p-value (t-test, $P(r\|\mathcal{M})$)	Avg. $P(r\|\mathcal{M})$ (Antarctic Deep Lake)	Avg. $P(r\|\mathcal{M})$ (Alaska Permafrost)
R09135	Tetrachloro-cis,cis-muconate <=>2,3,5-Trichlorodienelactone + Hydrochloric acid	1.0000	0.0363	0.1449	0.1588
R09137	2,3,5-Trichloromaleylacetate + 2 NADH + 2 H+ <=>2,4-Dichloro-3-oxoadipate + 2 NAD+ + Hydrochloric acid	0.0606	0.0174	0.0423	0.1353
R09138	2,3,5-Trichloromaleylacetate + 2 NADPH + 2 H+ <=>2,4-Dichloro-3-oxoadipate + 2 NADP+ + Hydrochloric acid	0.0606	0.0170	0.0423	0.1360
R09157	Trichloroacetate + 2 H2O <=>Oxalate + 3 Hydrochloric acid	1.0000	0.0205	0.5551	0.7350
R09223	2-Chloro-5-methylmaleylacetate + NADH + H+ <=>5-Methylmaleylacetate + NAD+ + Hydrochloric acid	0.0606	0.0181	0.0259	0.0822
R09224	5-Methylmaleylacetate + NADH + H+ <=>2-Methyl-3-oxoadipate + NAD+	0.0606	0.0215	0.0219	0.0675
R09229	3-Methyl-cis,cis-hexadienedioate <=>3-Methylmuconolactone	1.0000	0.0000	0.1707	0.1175
R09305	7,9,12-Octaketide intermediate 3 <=>Octaketide bicyclic intermediate + H2O	0.0606	0.0250	0.3333	1.0000
R09379	ATP + (R)-4-Phosphopantoate + beta-Alanine <=>AMP + Diphosphate + D-4'-Phosphopantothenate	0.0606	0.0250	1.0000	0.3333
